# Low Doses of β-Caryophyllene Reduced Clinical and Paraclinical Parameters of an Autoimmune Animal Model of Multiple Sclerosis: Investigating the Role of CB_2_ Receptors in Inflammation by Lymphocytes and Microglial

**DOI:** 10.3390/brainsci13071092

**Published:** 2023-07-19

**Authors:** Vahid Reza Askari, Vafa Baradaran Rahimi, Reza Shafiee-Nick

**Affiliations:** 1 International UNESCO Center for Health-Related Basic Sciences and Human Nutrition, Mashhad University of Medical Sciences, Mashhad 9177948564, Iran; 2 Applied Biomedical Research Center, Mashhad University of Medical Sciences, Mashhad 9177948564, Iran; 3 Department of Cardiovascular Diseases, Faculty of Medicine, Mashhad University of Medical Sciences, Mashhad 9177948564, Iran; baradaranrv@mums.ac.ir; 4 Department of Pharmacology, Faculty of Medicine, Mashhad University of Medical Sciences, Mashhad 9177948564, Iran; shafieenickreza@gmail.com

**Keywords:** Multiple Sclerosis, EAE, β-Caryophyllene, cannabinoid receptor, lymphocytes, microglia

## Abstract

Multiple Sclerosis (MS) is a prevalent inflammatory disease in which the immune system plays an essential role in the damage, inflammation, and demyelination of central nervous system neurons (CNS). The cannabinoid receptor type 2 (CB_2_) agonists possess anti-inflammatory effects against noxious stimuli and elevate the neuronal survival rate. We attempted to analyze the protective impact of low doses of β-Caryophyllene (BCP) in experimental autoimmune encephalomyelitis (EAE) mice as a chronic MS model. Immunization of female C57BL/6 mice was achieved through two subcutaneous injections into different areas of the hind flank with an emulsion that consisted of myelin Myelin oligodendrocyte glycoprotein (MOG)_35–55_ (150 µg) and complete Freund’s adjuvant (CFA) (400 µg) with an equal volume. Two intraperitoneal (i.p.) injections of pertussis toxin (300 ng) were performed on the animals on day zero (immunizations day) and 48 h (2nd day) after injection of MOG + CFA. The defensive effect of low doses of BCP (2.5 and 5 mg/kg/d) was investigated in the presence and absence of a CB_2_ receptor antagonist (1 mg/kg, AM630) in the EAE model. We also examined the pro/anti-inflammatory cytokine levels and the polarization of brain microglia and spleen lymphocytes in EAE animals. According to our findings, low doses of BCP offered protective impacts in the EAE mice treatment in a CB2 receptor-dependent way. In addition, according to results, BCP decreased the pathological and clinical defects in EAE mice via modulating adaptive (lymphocytes) and innate (microglia) immune systems from inflammatory phenotypes (M_1_/Th_1_/Th_17_) to anti-inflammatory (M_2_/Th_2_/T_reg_) phenotypes. Additionally, BCP elevated the anti-inflammatory cytokine IL-10 and reduced blood inflammatory cytokines. BCP almost targeted the systemic immune system more than the CNS immune system. Thus, a low dose of BCP can be suggested as a therapeutic effect on MS treatment with potent anti-inflammatory effects and possibly lower toxicity.

## 1. Introduction

Multiple Sclerosis (MS) is a prevalent inflammatory problem with special interfacing with the immune system that leads to inflammation and demyelination of neurons and axonal loss in the central nervous system (CNS) [1]. As categorized by the World Health Organization (WHO), MS is a debilitating problem that influences above one million patients globally, with a higher incidence among women (female/male ratio = 3/1) [1,2]. Despite the unclear pathophysiology mechanism of MS, some research works suggest that it is an organ-specific T-cell-mediated autoimmune disease [1,2,3]. Nevertheless, the effect of B-cells should not be neglected because B-cell targeted therapies may be successful in treating some patients with MS [1,2,3].

EAE is an important model commonly utilized to investigate new treatments for chronic MS disease [4,5]. In this model, similar to those seen in MS disease, CD4^+^ T helper (Th) cells that target myelin are the primary culprits for the onset, exacerbation, and progress of defects in EAE because transferring these cells to naïve mice induces EAE [4,5]. Previous works report T helper type 17 (Th_17_) and type 1 (Th_1_) cells as the primary population of Th cells in both EAE and patients with MS. Principally. These cells generate inflammatory cytokines. They can also activate microglia and develop neuroinflammation [6]. As one of their main characteristics, Th_1_ cells mainly generate TNF-α and IFN-γ, while Th_17_ cells mainly produce and release interleukin (IL)-17 and IL-22 [6,7]. On the contrary, regulatory T (T_reg_) and Th_2_ cells primarily generate and release the anti-inflammatory cytokines (TGF-β_1_, IL-10, IL-4) [6,7], which result in the inflammation resolution, microglia inactivation, and reduced progression of MS [4,5,6].

Recently, many studies have reported that as a result of the cannabinoid receptor type 2 (CB_2_) activation, many protective effects are provided against various exogenous and endogenous noxious stimuli by reducing the pro-inflammatory cytokine levels (IL-6, TNF-α, IFN-γ, and IL-17), and elevating the anti-inflammatory cytokine level (IL-10). Additionally, CB_2_ receptor activation propagates neuronal survival rate by raising the anti-inflammatory mediator levels [8,9,10]. Several reports also demonstrated that PPAR-γ activation is seemingly required to develop anti-inflammatory effects of endocannabinoids and cannabinoid agonists [8,11]. In this context, some studies have shown that the use of pharmacological PPARγ antagonist GW9662 or PPARγ siRNA substantially declined the promising protective effects of cannabinoid receptor (CBR) agonists [8,11]. As a joint characterization for G-protein coupled receptors, we should consider that long-term exposure to a selective CB2 agonist can activate non-canonical signaling pathways through the same receptor [12,13]. In the case of long-term activation or a high dose of CBR, the βγ-subunit of G-protein coupling-CBR activates sphingomyelinase (SMase), leading to an increment in production levels and accumulation of ceramide inside the cell [14,15,16]. Moreover, it has been shown that the activation of SMase and sphingosine kinase during stress increases the levels of ceramides and consequent metabolites, which may influence the Nrf2 and PPARγ signaling pathways [17,18].

β-Caryophyllene (BCP), a selective CB_2_ agonist (Ki = 155 ± 4 nM), is abundantly available in medicinal herbs, including *Origanum vulgare* L. *Piper nigrum* L., and *Syzygium aromaticum* [19,20]. There are two groups of studies regarding the protective effects of BCP, in which both high and low doses of BCP apply their antioxidant and anti-inflammatory impacts in a CB_2_ receptor-dependent manner because the use of an antagonist completely omits the effects of BCP [15,21,22]. In the first group, it was illustrated that low doses and concentrations of BCP (≤2 μM or 10 mg/kg) exert protective and healing effects, such as anti-inflammatory [19], analgesic [19], and decreasing neuropathic pain [23], as well as anti-apoptogenic effects against different noxious stimuli like glutamate-induced excitotoxicity [24] and MPP^+^ (1-methyl-4-phenylpyridinium)-induced Parkinson model [21], in a CB_2_ receptor-dependent manner. In the other group, it has been demonstrated that the higher concentrations and doses of BCP (≥10 µM and 25 mg/kg) exert protective effects, such as ameliorative properties in animal models of MS [25], neuropathic pain [20,26], ulcerative colitis [27], and β-Amyloid (Aβ)-induced inflammation and cytotoxicity in BV-2 murine microglia cell line [22].

According to our previous studies, the protective impacts of both high and low concentrations of BCP were CB_2_ receptor-mediated by functional-selectivity assay. In fact, we demonstrated that all BCP concentrations decreased the cyclic adenosine monophosphate (cAMP) level in a concentration-dependent manner, and that this effect depended on the CB2 receptor because a selective pharmacological antagonist (AM630) provided a right-ward shift [14,15]. Based on our results regarding the protective effects of low BCP concentrations and their higher potency and also efficacy than a high concentration of BCP [14,15], we hypothesized that the low dose of BCP probably exerts curative and ameliorative effects in the MS treatment. Thus, the protective effects of low doses of BCP were investigated with and without a selective pharmacological CB_2_ receptor antagonist (AM630) in EAE as a chronic MS model. Experimentally, the polarizations of microglia (M_1_/M_2_) and lymphocytes (Th_1_/Th_2_ and Th_17_/T_reg_), and the profiles of anti-inflammatory and inflammatory cytokine levels were examined in EAE mice.

## 2. Materials and Methods

### 2.1. Chemicals and Kits

Sigma–Aldrich Chemical Co. (St. Louis, MO, USA) provided the following: RPMI-1640 and DMEM culture media, β-Caryophyllene (BCP, C9653 SIGMA), penicillin plus streptomycin (pen/strep), amphotericin B, dimethyl sulfoxide (DMSO), FBS, Ficoll^®^, dispase II, 2′, 7′-dichlorofluorescein diacetate (DCFH DA, code D6883), DNAse I, Griess reagent (G4410 SIGMA), sulfosalicylic acid, potassium phosphate buffer, Nonidet P40, Bradford reagent (B6916 SIGMA), sulfanilamide, triton-X, dithiothreitol (DTT), n-(1-naphthyl) ethylenediamine, EDTA disodium salt, 4-(2-hydroxyethyl)-1-piperazineethanesulfonic acid (HEPES), protease inhibitor cocktail, potassium chloride (KCl), phenylmethanesulfonyl fluoride (PMSF), pertussis toxin, complete Freund’s adjuvant (CFA, H37Ra strain), HBSS and other materials for culturing cell. Santa Cruz Biotechnology Inc. (Baghdad, Iraq) provided AM-630 (sc-200365A) as a selective CB_2_ cannabinoid antagonist. ELISA kits, including IL-6 (BMS603-2, TNF-α (BMS607-3, 3.7 pg/mL), 6.5 pg/mL), IL-17 (BMS6001, 1.6 pg/mL), IL10 (BMS614INST, 5.28 pg/mL), IL-4 (BMS613HS, 0.32 pg/mL), TGF-β_1_ (BMS608-4, 7.8 pg/mL), and IFN-γ (BMS606, 5.3 pg/mL) were prepared from eBioscience (San Diego, CA, USA). The transcription factors forkhead box P3 (Foxp3, MBS731757, 0.1 ng/mL), T-box transcription factor TBX21 (T-bet, MBS763917, 0.094 ng/mL), retinoic acid receptor (RAR)-related orphan receptor gamma (ROR-γt, MBS751809, 0.1 ng/mL), arginase-1 (Arg-1, MBS1601158, 0.28 ng/mL) and inducible nitric oxide synthase (iNOS, MBS764477, 0.188 ng/mL) mouse ELISA kits were prepared by MyBioSource (San Diego, CA, USA). Mouse prostaglandin E2 (PGE_2_, CSB-E07966m, 0.2 pg/mL) ELISA kit was obtained from CUSABIO (Wuhan, China). Salari Institute of Cognitive and Behavioral Disorders (SICBD), Iran, provided Myelin oligodendrocyte glycoprotein 35–55 (MOG_35–55_, sequence: MEVGWYRSPFSRVVHLYRNGK). The BrdU kit (colorimetric) was bought from Roche Applied Science (Indianapolis, IN, USA). Biolegend (San Diego, CA, USA) provided RBC Lysis Buffer (10×, Cat No:420301).

### 2.2. Animals and Husbandry

Salari Institute of Cognitive and Behavioral Disorders (SICBD) provided female C57BL/6 mice (18–20 g, 8–10 weeks old). Afterward, the mice were maintained in the animal lab of the Department of Pharmacology, Faculty of Medicine, Mashhad University of Medical Sciences. The room temperature where the mice were kept was controlled (22–25 °C), and 50% relative humidity and a 12/12 h light/dark cycle were applied. Animals all could freely access commercial standard rodent chow and safe and clean filtered tap water. In all procedures and experiments, the *NIH’s Guideline for the Care and Use of Laboratory Animals* was approved by the *Animal Ethics Committee of Mashhad University of Medical Sciences* (No. 960037, IR.MUMS.fm.REC.1396.200).

### 2.3. Induction of EAE and Clinical Evaluation

**Figure 1:**
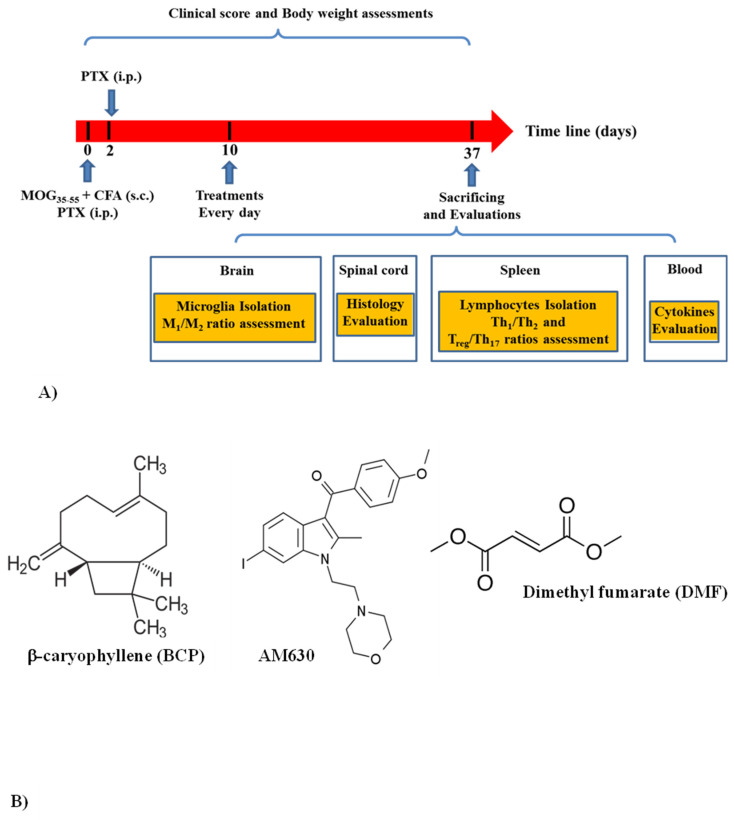
Treatment protocol, chemical structures, and flow cytometric analysis of cell-surface expression of receptors; (**A**) the summarized protocols of treatment and induction; (**B**) chemical configurations of the compounds used in the research.

Immunization of female C57BL/6 mice was achieved through two subcutaneous injections into different areas of the hind flank with an emulsion that consisted of myelin MOG_35–55_ (150 µg) and CFA (400 µg) with an equal volume. Two intraperitoneal (i.p.) injections of pertussis toxin (300 ng) were performed on the animals on day zero (immunization day) and 48 h (2nd day) after injection of MOG + CFA in the Salari Institute of Cognitive and Behavioral Disorders (SICBD). The EAE induction procedure is summarized in Figure 1A. The weight of mice was obtained daily, and clinical scoring was performed from day 0 to day 37 (the experiment’s last day). The procedure of clinical scoring was performed with the help of two independent researchers so that one of them was blind to the process of experiments and also grouping. The descriptions of Bittner et al. [28] were followed in the clinical scaling score of EAE, which is presented in Table 1. Also, animals were examined for the MDO (days after immunization), incidence (sick/total), MMCS (during the whole treatment course), CDI (total disease score throughout the experiment), and MCS (at the peak day).

### 2.4. Experimental Group and Study Design

#### 2.4.1. Experiment One

The protocol was designed to evaluate the protective effects of low BCP doses (2.5, 5 mg/kg) in the EAE model (Table 2). To examine the treatment effects of BCP (2.5 and 5 mg/kg doses), BCP (diffused in corn oil) was used as oral administration once per day from ten days after immunization, showing significant increases in the clinical score compared to the sham group (n = 8/each group, Figure 1A). The BCP doses were selected given our earlier in vitro tests concerning the BCP (oligodendrocytes [15] and microglia [14]) and earlier in vivo surveys concerning the BCP’s protective effects (25, 50 mg/kg/day [25,26]) in EAE. Accordingly, we carried out our experiments with one-tenth of the doses that were previously for BCP. DMF was regarded as a positive control group. It is a standard used orally, and the FDA has proven it for MS patients. DMF-group took 30 mg/kg DMF (diffused in 0.08% *w*/*v* methylcellulose) that was received twice daily per oral gavage (n = 8) [29,30]. In the control group, EAE mice received identical vehicle volume (n = 8). The sham group had normal mice receiving the same amount of PBS injections rather than EAE induced by MOG_35–55_.

#### 2.4.2. Experiment Two

This procedure was performed to evaluate the possible role of the CB_2_ receptor in the protective effects of BCP (Table 2). AM630 (1 mg/kg; i.p., [31,32,33], dissolved in 2% DMSO), as a pharmacological CB_2_ receptor antagonist, was administrated 30 min before the BCP treatment (5 mg/kg; p.o., n = 8, showed the best protection in experiment one). As the control group, EAE mice took the identical volume of all vehicles, including neither BCP nor AM630 medications (n = 8). The dose of AM630 as a highly selective antagonist of the CB_2_ receptor (Ki = 31.2 nM) was chosen based on previous studies indicating that the dose of 1 mg/kg of AM630 was pharmacologically optimal, devoid of any in vivo toxicity and also exerted no behavioral and molecular effects when used alone [31,32,33].

### 2.5. Histological Evaluation

When sacrificing (day 37), in order to evaluate the CNS inflammation level, xylazine (10 mg/kg) and ketamine (100 mg/kg) were used for the deep anaesthetization, and then they were sacrificed. Then, isolation of the spinal cord was performed, and it was kept in 4% (*v*/*v*) paraformaldehyde. Hematoxylin and Eosin (H&E) were used for staining Longitudinal sections (5 μm thick) of the spinal cords’ paraffin-embedded to evaluate inflammation. Afterward, Sections were scored by light microscopy in a blinded way (by analyzing three fields) at 100× magnification (Olympus, Tokyo, Japan). Based on the previous works, the pathological scores were obtained as follows: 0—no inflammation; 1—low inflammatory cell levels; 2—perivascular infiltrates; and 3—the extended intensity of perivascular cuffing with expanding to contiguous tissue.

### 2.6. Serum Preparation and Inspection of the Cytokines Profile

On day 37 (the last day of the experiment), after the deep anesthesia, using cardiac puncture, 1.5 mL of blood was taken and collected in a 2 mL EDTA-coated microtube that contained aprotinin (140 µg). The samples were immediately centrifuged for 10 min at 4 °C at 2500× *g*. Then, we collected the plasma and kept it at −20 °C for the next experiment. The anti-inflammatory (IL-10) and inflammatory (IL-17, TNF-α, IL-6) cytokine levels were specified by the ELISA kits considering the instruction of the manufacturer, and were presented as pg per mL of plasma.

### 2.7. Separation of Lymphocyte from Spleen and Cell Culture

After the euthanasia of animals, we eliminated the spleen and put it into a tube (15 mL) with 5 mL ice-cold RPMI-1640/FBS (RPMI-1640, streptomycin (100 µg/mL), penicillin (100 U/mL), 2% *v*/*v* FBS). The spleen was then cut into slices by ice-cold RPMI-1640/FBS in the 75 µm cell strainer mesh to provide a high-quality cell suspension. The resulting cell suspension was centrifuged for 10 min at 4 °C at 45. Then, the supernatant was eliminated without disturbing the pellet [34]. Thereafter, sterilized PBS (15 mo) was mixed with the cell pellet, followed by pouring the suspension onto Ficoll^®^ (15 mo) in a 50 mL sterilized conical tube. It was then centrifuged at 400 g for 20 min at a controlled temperature (22–25 °C). After carefully removing the buffy coat layer, it was washed with buffer phosphate saline thrice for 10 min at 130 g. In order to eliminate the remaining red blood cells, we co-cultured the cells with 1× diluted RBC lysis buffer (10×) based on the procedure proposed by the manufacturer, and the supernatant was aspirated without agitating the pellet. To exclude monocytes, pellets were put into a supplemented RPMI-1640 (100 U/mL penicillin, 2 mM L-glutamine, 10% FBS, 1 mM sodium pyruvate, 100 µg/mL streptomycin, HEPES buffer), followed by culturing in flasks (250 mL) for one night. Then, the supernatant, with lymphocytes, was placed into a sterilized conical tube (50 mo) [34,35]. Isolated lymphocytes were cultured in RPMI-1640 enriched with 10% *v*/*v* of heat-inactivated FBS, 1% *v*/*v* of pen/strep (100×), 2 mM L-glutamine, and 0.5 µg/mL amphotericin B. Cells were kept at 37 °C in a humidified incubator and 5% *v*/*v* CO_2_.

#### 2.7.1. BrdU Cell Proliferation Assay

For culturing Lymphocytes, 96-well plates were used (5 × 10^4^ cells/well), and their treatment was performed with medium + MOG_35–55_ peptides (30 μg/mL, [36]) or medium alone at 37 °C for 48 h and 5% *v*/*v* CO_2_. In the last 24 h, BrdU labeling solution (0.1% *v*/*v*) was mixed with the plates. Considering the manual provided by the manufacturer, a cell proliferation ELISA BrdU kit (colorimetric) was used to assess cell proliferation. The microplate reader was used for reading the absorbance at 450 nm. The mean cell proliferation level of the MOG_35–55_ shame group was 100%.

#### 2.7.2. Examination of the Cytokines Profile

For providing a deeper understanding of the impact of various treatments on the Th_17_/T_reg_ ad Th_1_/Th_2_ polarization in EAE, the Th_1_ (IFN-γ), Th_2_ T_reg_ (IL-10, IL-4, TGF-β_1_), and Th_17_ (IL-17) levels were evaluated related to cytokines. In addition, inflammatory cytokines TNF-α and IL-6 were also assessed in the supernatant of lymphocytes stimulated by MOG_35–55_. Thus, 12-well plates were used for culturing the isolated lymphocytes (2 × 10^6^ cells/well), and they were treated with medium+MOG_35–55_ peptides (30 μg/mL) or medium alone at 37 °C for 48 h and 5% *v*/*v* CO_2_. Then, after collecting the supernatants, they were maintained at −20 °C to evaluate the cytokine levels using the ELISA technique. The cytokine levels were standardized and presented as pg/mg protein. Bradford’s method was used to measure each experiment’s total protein content [37].

#### 2.7.3. Evaluation of Intracellular Levels of Transcription Factors

In order to confirm the polarization of lymphocytes, the levels of valid and specific transcription factors for Th_1_ (T-bet), Th_2_ (GATA3), T_reg_ (Foxp3), and Th_17_ (ROR-γt) cells were assessed [5,38]. To this end, we cultured the separated lymphocytes (2 × 10^6^ cells/well) in plates with 12-wells, followed by treatment with medium + MOG_35–55_ peptide (30 μg/mL, [36]) or medium alone at 37 °C for 48 h and 5% *v*/*v* CO_2_. Then, lymphocytes were centrifuged to provide the pellets, and they were rinsed three times with PBS. The pellets were then suspended again into the cell lysis buffer (10 mM HEPES; 10 mM KCl, pH 7.5, 0.1 mM EDTA, 0.5% Nonidet-P40, 1 mM DTT, 0.5 mM PMSF with the protease inhibitor cocktail) and kept for 15–20 min with recurrent vortexing. Also, total protein contents were obtained using Bradford’s method [37]. We stored separated proteins at −20 °C to evaluate the transcription factors’ levels according to the ELISA procedure given in the manufacturer’s guide. The levels of transcription factors were presented as pg/mg or ng protein.

### 2.8. Microglia Isolation

The approach provided by Lee and Tansey [39,40,41] with some modifications was used for isolating Microglial cells. After mice decapitation, mice brains were separated from meanings, undergoing enzymatic and mechanical dissociations by papain (1 mg/mL as the end concentration), dispase II (1.2 U/mL as the end concentration), and DNAse I (20 U/mL as the end concentration). The sliced brain was placed in a 15 mL tube for 20 mon, which contained the enzymes (3 mL) in the cell culture incubator. Then, the enzymes were neutralized with a complete medium (5 mL), and the cells were centrifuged at 250× *g* for 5 min at a controlled temperature. Following this process, the supernatants were eliminated. The pellets were suspended again into 4 mL per brain of 37% SIF (9 parts of Ficoll with 1 part 10× HBSS) and poured onto 4 mL of 70% SIF. A total of 30% SIF (4 mL) was pipetted slowly over the 37% SIF layer, and then 2 mL of HBSS was added. The mix was centrifuged at 300× *g* for 40 min at 18 °C with no brake. A sterilized 15-mL conical tube was used for collecting the 70–37% interphase. A total of 6 mL of HBSS per 2 mL of the obtained interphase was poured into the tube to the interphase Ficoll was thinned and centrifuged at 4 °C at 500× *g* for 7 min. Isolated microglia cells were cultured in the enriched DMEM (10% *v*/*v* of heat-inactivated FBS, 1% *v*/*v* of pen/strep (100×), 0.5 µg/mL amphotericin, 2 mM L-glutamine). Cells were maintained in a humidified incubator at 5% *v*/*v* CO_2_ and 37 °C.

#### 2.8.1. Cell Proliferation Assay

Following culturing Microglia cells (8 × 10^3^ cells/well) in 96-well plates, their treatment was performed with medium + MOG_35–55_ peptide (30 μg/mL, [36]) or medium alone at 37 °C for 48 h at 5% *v*/*v* CO_2_. In the last 24 h, we added BrdU labeling solution (0.1% *v*/*v*) to the plates. A cell proliferation ELISA-based BrdU kit (colorimetric) was used based on the manufacturer’s guide for assessing cell proliferation. The microplate reader was utilized for reading the absorbance at 450 nm. The mean cell proliferation level of the MOG_35–55_ shame group was 100%.

#### 2.8.2. Evaluation of the Cytokine Levels

The ELISA-based technique was used to measure levels of inflammatory and anti-inflammatory (IL-10) and (PGE_2_, TNF-α cytokines. After culturing the cells in plates with 6-wells (10^6^ cells/per well), they were incubated with medium + MOG_35–55_ peptides (30 μg/mL, [36]) or medium alone at 37 °C for 48 h in a 5% *v*/*v* CO_2_ incubator. Lastly, the levels of cytokines were measured by collecting the supernatants. The cytokine levels were presented as pg/mg protein.

#### 2.8.3. Evaluation of the Intracellular Levels of Arg-1 and iNOS

Considering the manufacturer’s guide, a commercial ELISA-based kit was used to assess Intra-cellular levels of Arg-1 and iNOS (as indexes of M2 and M1 cells). Following culturing the cells in a 6-well plate (2 × 10^6^ cells/each well), they were incubated with medium+MOG_35–55_ peptides (30 μg/mL, [36]) or medium alone at 37 °C for 48 h in a 5% *v*/*v* CO_2_ incubator. The cells were collected and lysed by the use of a lysis buffer. Afterward, the cells were homogenized (DIAX 100, Germany) for 2–3 min in the cold water (0–4 °C) with vortexing (every 30 s). The cells were centrifuged for 10 min at 12,000× *g* at 4 °C, and 50 µL of supernatants were evaluated. The Arg-1 and iNOS levels were presented as ng/mg protein.

#### 2.8.4. Evaluation of Urea and NO Levels

The supernatants of cultured microglia were used for measuring the levels of urea (generated by M_2_ cells) and NO metabolites (generated by M_1_ cells). The Griess technique [42] was used to examine the NO production level. Briefly, we incubated 50 μL of the supernatant with an identical volume of *n*-(1-naphthyl)-ethylenediamine and sulfanilamide in 2N hydrochloric acid for 10 min at the controlled temperature. A microplate reader was used for reading the absorbance at 540 nm. By the use of the sodium nitrite standard curve [43,44], the NO concentration was specified.

A commercial urea assay kit was used to determine urea levels. Shortly after, 50 μL of supernatant and equal volumes of medium (blank) and 50 µL urea-solution (5 mg/dL or 850 μM) were mixed and incubated with the 200 µL working reagent for 50 min at a controlled temperature. The Stat Fax 2100 Awareness microplate reader was utilized for reading the absorbance at 430 nm. The urea and NO levels were given as nmol/mg protein [43,44].

### 2.9. Statistical Analysis

GraphPad Prism^®^ 6 software (San Diego, CA, USA) was employed for data analysis, and data were presented in terms of their none-parametric or parametric nature as median ± range (for pathological score) or means ± SEM. For parametric data, normality tests were conducted based on Bartlett’s and Kolmogorov–Smirnov tests, which examine the homogeneity of variances. In case of passing the test, two groups were compared using an ANOVA test, following Dunnett’s *post hoc* multiple comparisons test. Following Tukey’s multiple comparison tests, a two-way ANOVA test was performed for body weight and a clinical score. Moreover, Kruskal–Wallis and Dunn’s post hoc multiple comparisons tests analyzed no parametric data. *P*-values (*p*) were statistically at *p* ≤ 0.05, 0.01, and 0.001. There was compliance between statistical analysis and data with the instructions on experimental design, analysis [45], data presentation, and sharing in preclinical pharmacology [46,47].

## 3. Results

### 3.1. BCP Effect on Body Weight and the Clinical Score of EAE Mice

**Figure 2:**
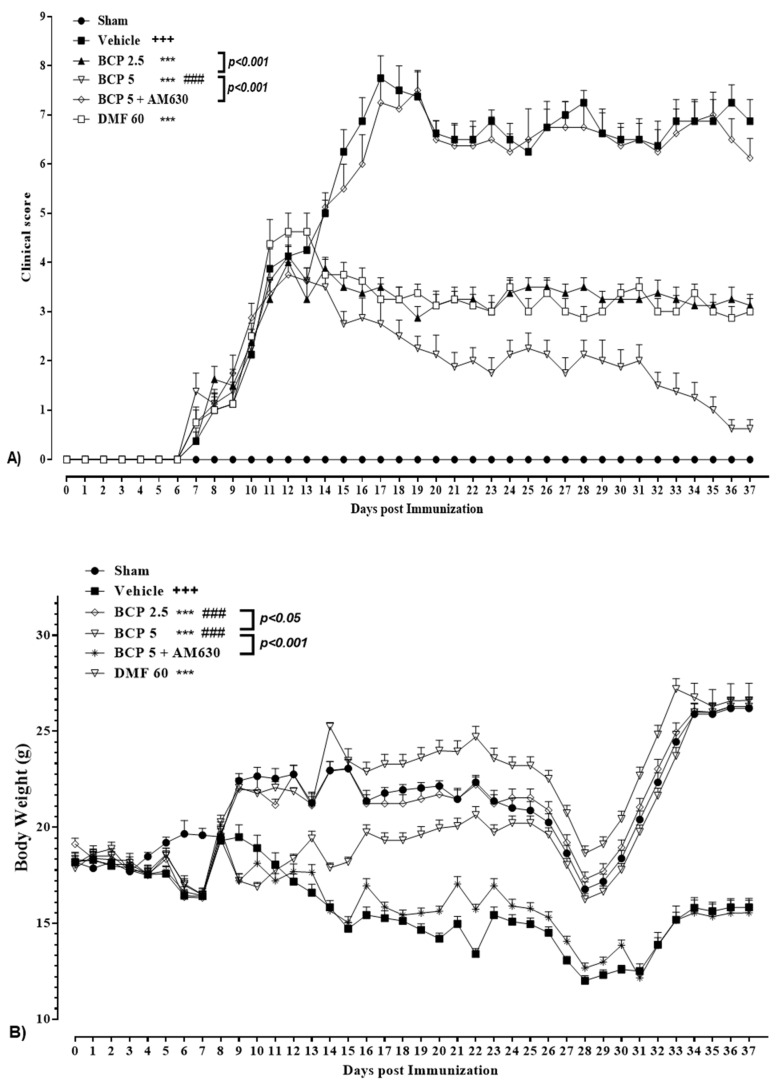
**The effects of BCP treatment on body weight and clinical score of EAE mice;** (**A**) The effects of DMF (60 mg/kg; 30 mg/kg twice a day; p.o.), BCP (2.5 and 5 mg/kg/day; p.o.), and the combination of a CB_2_ antagonist AM630 (1 mg/kg; i.p.) and BCP (5 mg/kg/day; p.o.) on the clinical score of EAE mice. (**B**) The effects of DMF (60 mg/kg; 30 mg/kg twice a day; p.o.), BCP (2.5 and 5 mg/kg/day; p.o.), and the combination of a CB_2_ antagonist AM630 (1 mg/kg; i.p.) and BCP (5 mg/kg/day; p.o.) on the body weight of EAE mice. Data were expressed as mean ± SEM, n = 8 for experiment protocols. A two-way ANOVA test was performed with the following Tukey’s multiple comparisons tests. (**^+^**) makes a comparison between the vehicle and sham groups, **^+++^**: *p* < 0.001.; (*) indicates the comparison with the vehicle group, ***: *p* < 0.001; (**^#^**) makes a comparison between BCP and the DMF group in each graph, **^###^**: *p* < 0.001.

As illustrated in Figure 2, the clinical score level of the vehicle EAE group showed a significant elevation in comparison to the sham group (*p* < 0.001). DMF treatment (60 mg/kg; p.o.) notably decreased the clinical score level compared to the vehicle group (*p* < 0.001, Figure 2A). BCP (2.5–5 mg/kg/d; p.o.) markedly reduced the clinical score level in EAE mice compared to the vehicle group in a dose-dependent fashion (*p* < 0.001 for both modes, Figure 2A). The BCP (5 mg/kg) group showed a significantly lower clinical score level compared to the DMF group (60 mg/kg) (Figure 2A). BCP effect on the clinical score level in EAE in the presence of AM6330 (1 mg/kg; i.p.) was totally abrogated (*p* < 0.001, Figure 2A).

As reported in Table 3, the vehicle group developed EAE, showing the initial signs of the onset of disease with a partially limp tail on day 6.72 ± 0.42, attaining an MCS of 7.75 ± 0.47 on day 17. In the BCP-treated group (2.5 and 5 mg/kg/d), the incidence of the disease was decreased to 58.3 and87.5% (*p* < 0.001), and disease severity was markedly attenuated, with MCS 3.75 ± 0.18 (*p* < 0.001) and 2.75 ± 0.43 (*p* < 0.001) on day 17 compared to the vehicle group (7.75 ± 0.47), respectively (Table 3). In addition, DMF (60 mg/kg/d, positive control) decreased the disease incidence to 87.5% and also significantly reduced the MCS to 3.43 ± 0.31 (*p* < 0.001) on day 17 in comparison with the vehicle group (Table 3). As illustrated by our findings, the concomitant use of AM630 (1 mg/kg/d) along with BCP (5 mg/kg/day) resulted in the effects of BCP (5 mg/kg) on disease incidence and MCS on day 17 were completely reversed (*p* < 0.001 for both cases) and did not present a significant difference with the vehicle group (Table 3).

The body weight of vehicle-treated EAE mice reflected a significant decline in comparison with the sham group (*p* < 0.001, Figure 2B). Treating with BCP (2.5 and 5 mg/kg/d) or DMF (60 mg/kg/d) caused a significant elevation in the body weight of EAE mice compared to the vehicle group (*p* < 0.001 for all modes, Figure 2B). The body weight of the BCP group (2.5, 5 mg/kg) was significantly more significant than the DMF group in EAE mice (*p* < 0.001 for all modes, Figure 2B). In the AM630 presence (1 mg/kg), the BCP effect (5 mg/kg) on the body weight was considerably eliminated compared to the BCP-treated (5 mg/kg) alone group (*p* < 0.001, Figure 2B).

### 3.2. The BCP Effects on the Level of the Histopathological Score in EAE Mice

**Figure 3:**
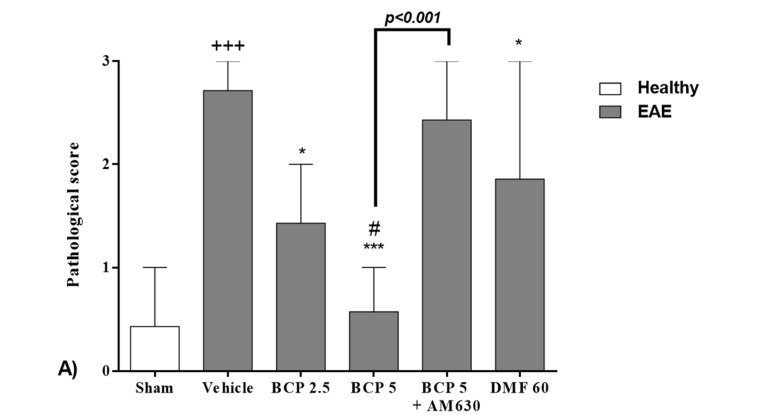

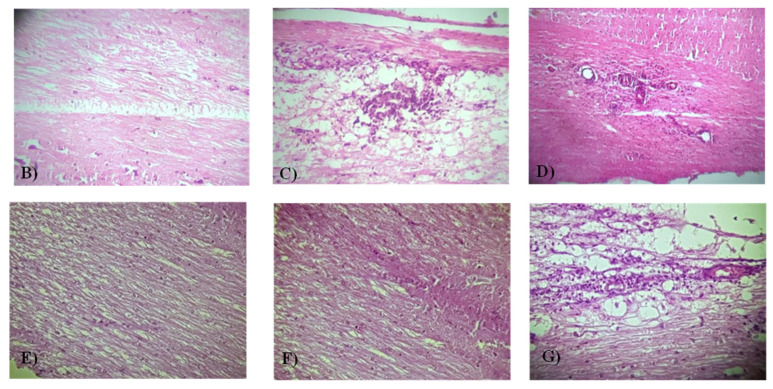
The effects of treatment with BCP on the inflammatory cell infiltration into the spinal cords and pathological score with H&E for enumerating infiltrating leukocytes; (**A**) The effects of DMF (60 mg/kg; 30 mg/kg twice a day; p.o.), BCP (2.5, 5 mg/kg/day; p.o.), and the combination of a CB_2_ antagonist AM630 (1 mg/kg; i.p.) with BCP (5 mg/kg/day; p.o.) with BCP (5 mg/kg/day; p.o.) on the pathological score in EAE mice. (**B**) Sham group. (**C**) Vehicle. (**D**) DMF 60 mg/kg. (**E**) BCP 2.5 mg/kg. (**F**) BCP 5 mg/kg. (**G**) BCP 5 mg/kg + AM630; indicates lumbar spinal cord parts of H&E staining (100×); 0—without inflammation; 1—low inflammatory cell levels, 2—perivascular infiltrates; and 3—the extended intensity of perivascular cuffing with extension into contiguous tissue. Data were presented as mean ± range, n = seven animals per group, three to four fields of view per animal for each experiment protocol. Considering that data has non-parametric nature, data analysis was performed using the Kruskal–Wallis test and Dunn’s post hoc multiple comparisons test. (*) indicates the comparison between varying doses of BCP and DMF groups and the vehicle group, *: *p* < 0.05, and ***: *p* < 0.001.; (^+^) makes a comparison between the vehicle and sham groups, ^+++^: *p* < 0.001.; ^#^ makes comparisons between BCP to DMF group in each graph, ^#^: *p* < 0.05.

The present study noted a prominent and massive leukocyte infiltration with multiple foci of inflammation in the vehicle group compared with the sham group (*p* < 0.001, Figure 3A,C). In DMF-treated (60 mg/kg/d) group, inflammation, and leukocyte infiltration were lower than in the vehicle group (*p* < 0.05, Figure 3A,D). BCP treatment (2.5 and 5 mg/kg/d) caused a significant decline in the level of the pathological score and the number of inflammatory cell infiltration into the spinal cord compared to the vehicle group (*p* < 0.05 and *p* < 0.001, Figure 3A,E,F). Nevertheless, the two doses of BCP did not show a significant difference. The addition of AM630 (1 mg/kg/d) before BCP (5 mg/kg/d) caused a considerable leukocyte infiltration into the spinal cords with multiple foci of inflammation compared to the BCP-treated alone group(5 mg/kg/d) (*p* < 0.001, Figure 3A,G), and no difference was noted compared to the vehicle-treated group.

### 3.3. The BCP Effects on Serum Levels of Cytokines in EAE Mice

**Figure 4:**
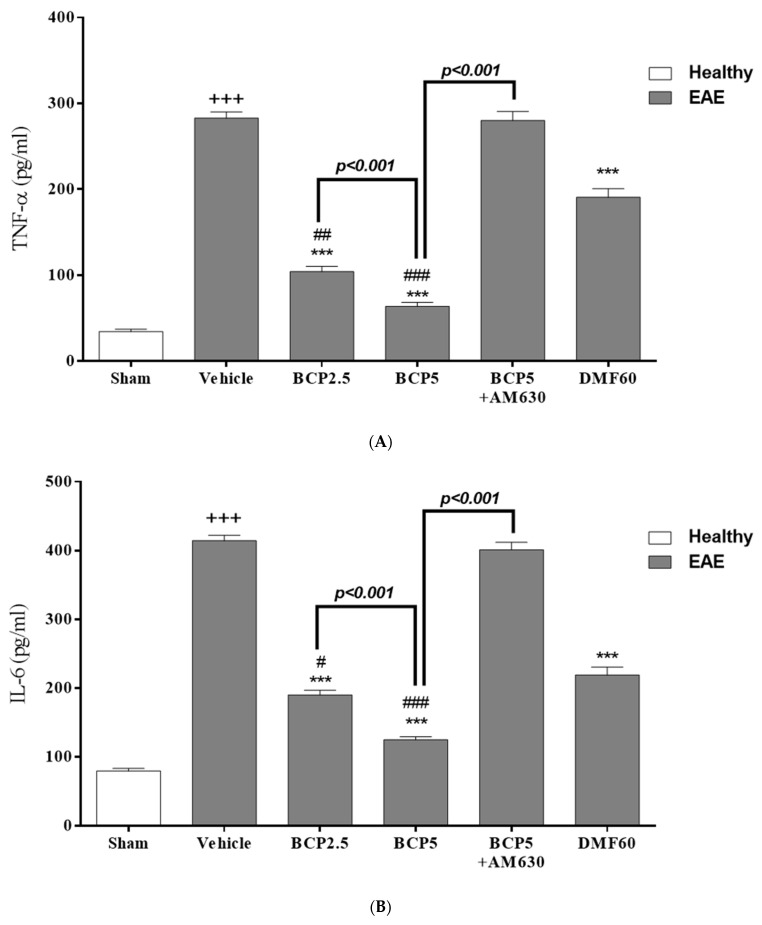

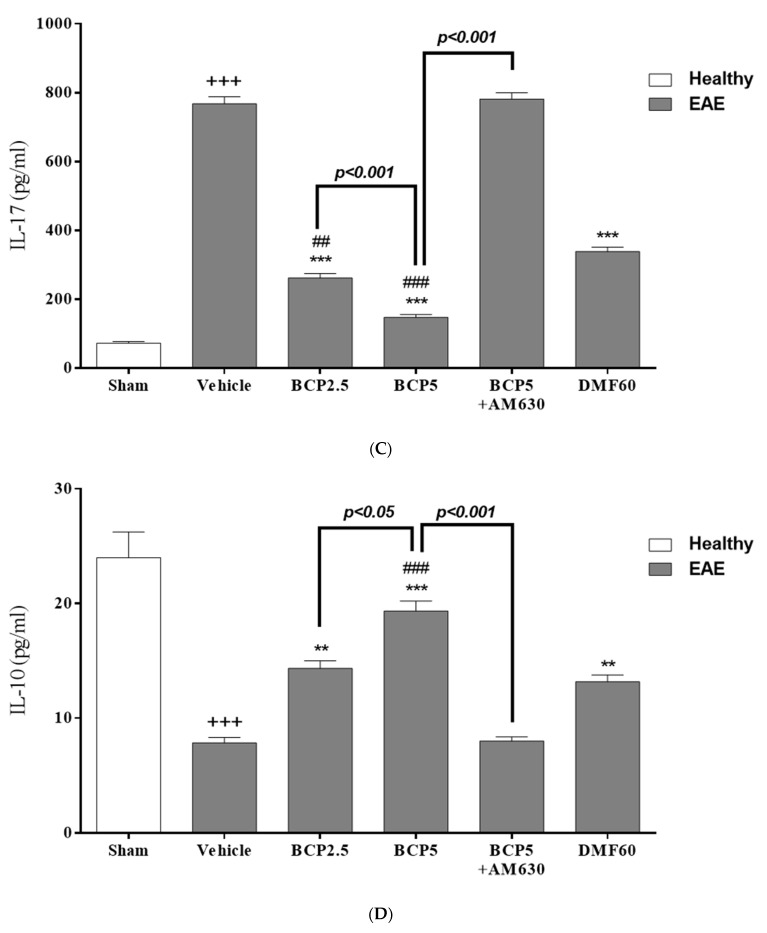

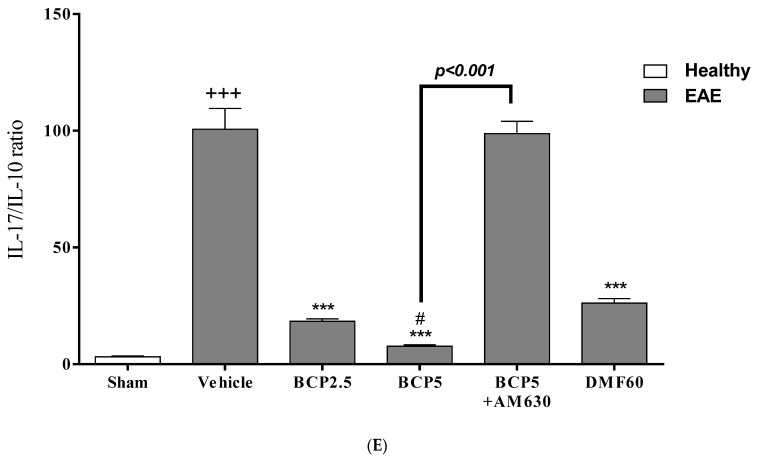
The effects of DMF (60 mg/kg; 30 mg/kg twice a day; p.o.), BCP (2.5, 5 mg/kg/day; p.o.), and the combination of a CB_2_ antagonist AM630 (1 mg/kg; i.p.) with BCP (5 mg/kg/day; p.o.) on the levels of anti-inflammatory and pro-inflammatory cytokines in the EAE mice serums; (**A**) TNF-α, (**B**) IL-6; (**C**) IL-17; (**D**) IL-10; and (**E**) IL-17/IL-10 ratio. Data were presented as Mean ± SEM, n = 6 animals in each group per experiment protocol. Two-way ANOVA and Tukey’s multiple comparisons test were performed. (*) indicates the comparison between varying doses of BCP and DMF groups to the vehicle group, **: *p* < 0.01, and ***: *p* < 0.001; (^+^) makes comparisons between the vehicle and sham groups, ^+++^: *p* < 0.001; ^#^ in comparison to DMF group in each graph, ^#^: *p* < 0.05, ^##^: *p* < 0.01, and ^###^: *p* < 0.001.

According to our findings, the inflammatory cytokine levels, including TNF-α (*p* < 0.001, Figure 4A), IL-6 (*p* < 0.001, Figure 4B), IL-17 (*p* < 0.001, Figure 4C), and IL-17/IL-10 ratio (*p* < 0.001, Figure 4E) were significantly higher in the vehicle-treated group compared to the sham group. On the contrary, the IL-10 level (*p* < 0.001, Figure 4D) was significantly lower in the vehicle group compared to the sham group. The levels of IL-6 (*p* < 0.001, Figure 4B), TNF-α (*p* < 0.001, Figure 4A), IL-17 (*p* < 0.001, Figure 4C), and IL-17/IL-10 ratio (*p* < 0.001, Figure 4E) in DMF-treated group (60 mg/kg) were considerably reduced, and the IL-10 level (*p* < 0.01, Figure 4D) was markedly elevated compared to the vehicle group.

Furthermore, our findings indicated that BCP (2.5, 5 mg/kg) generated a significant decline in the TNF-α levels (*p* < 0.001 for both modes, Figure 4A), IL-6 (*p* < 0.001 for both modes, Figure 4B), and IL-17 (*p* < 0.001 for both modes, Figure 4C), and IL-17/IL-10 ratio (*p* < 0.001 for both modes, Figure 4E) compared with the vehicle group. The IL-10 level in the BCP-treated group (2.5, 5 mg/kg/d) was meaningfully improved compared with the vehicle group (*p* < 0.01 for BCP 2.5 mg/kg, and *p* < 0.001 for BCP 5 mg/kg, Figure 4D). We observed that there is a meaningful difference between the effects of 2.5 and 5 mg/kg of BCP in EAE mice (*p* < 0.001 for TNF-α, IL-6, and IL-17, and *p* < 0.05 for IL-10, Figure 4A–D). In the presence of AM630 (1 mg/kg), the effects of BCP (5 mg/kg) on all measured parameters were utterly reversed compared with the BCP-treated alone group (5 mg/kg) (*p* < 0.001 for all modes, Figure 4A–E), and did not differ to vehicle group.

### 3.4. The Effects of BCP on Cell Proliferation and Cytokines Levels of Spleen Lymphocytes of EAE Mice with and without MOG Stimulation

**Figure 5:**
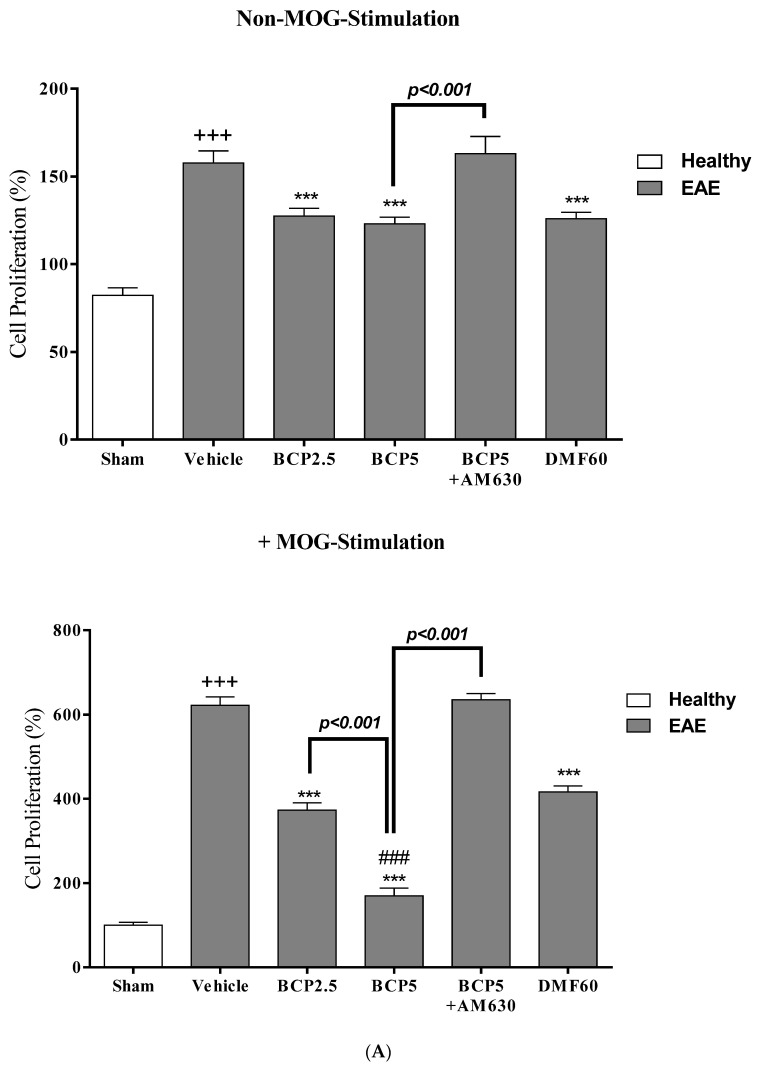

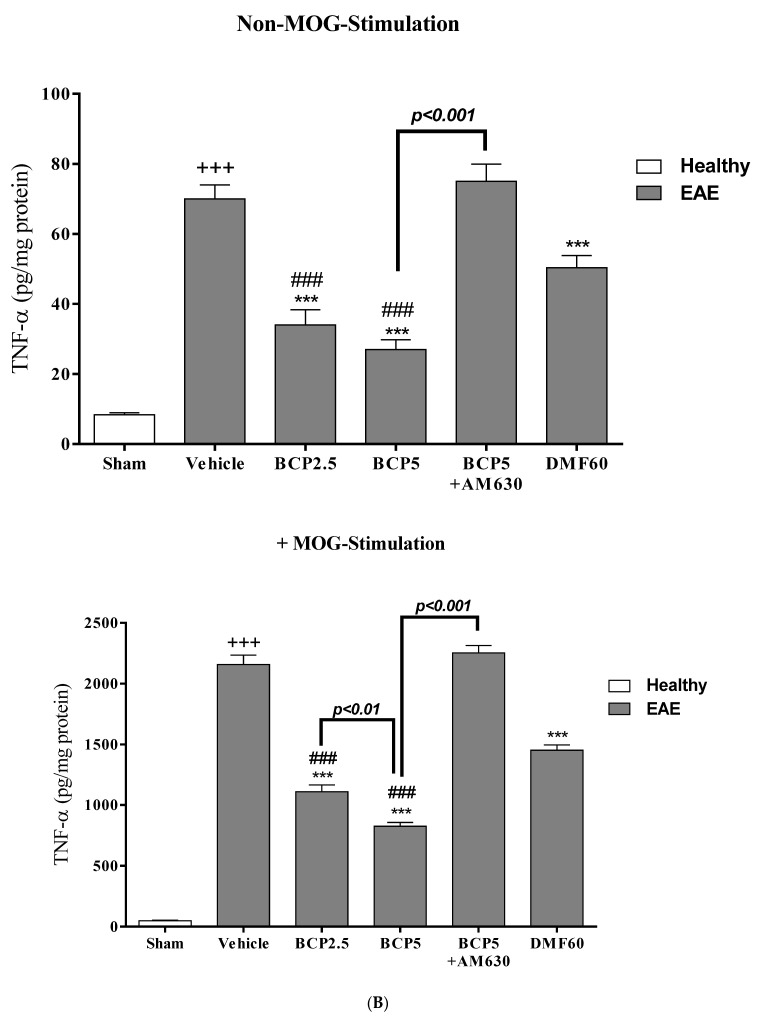

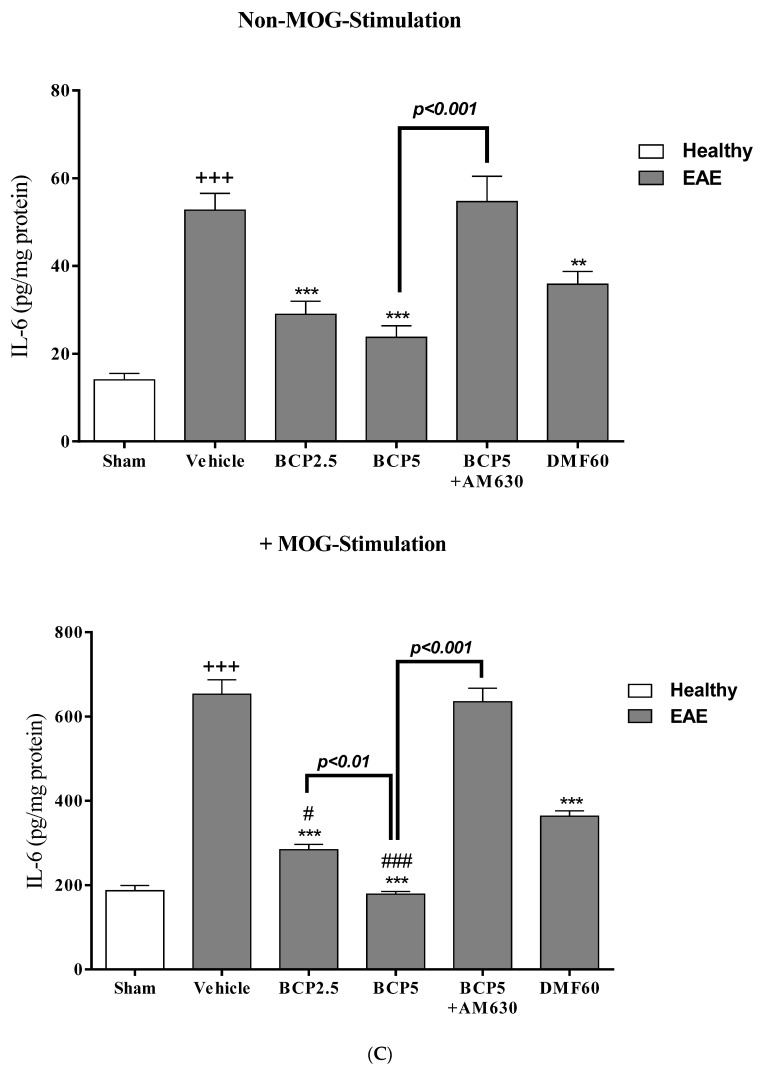

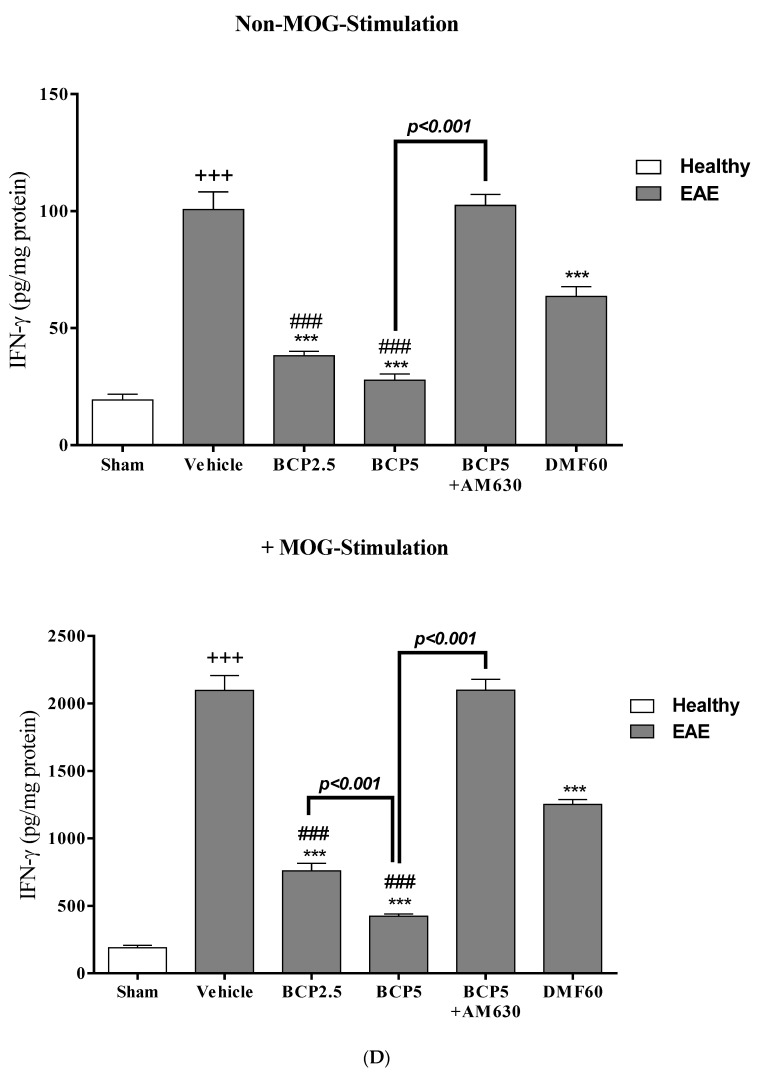

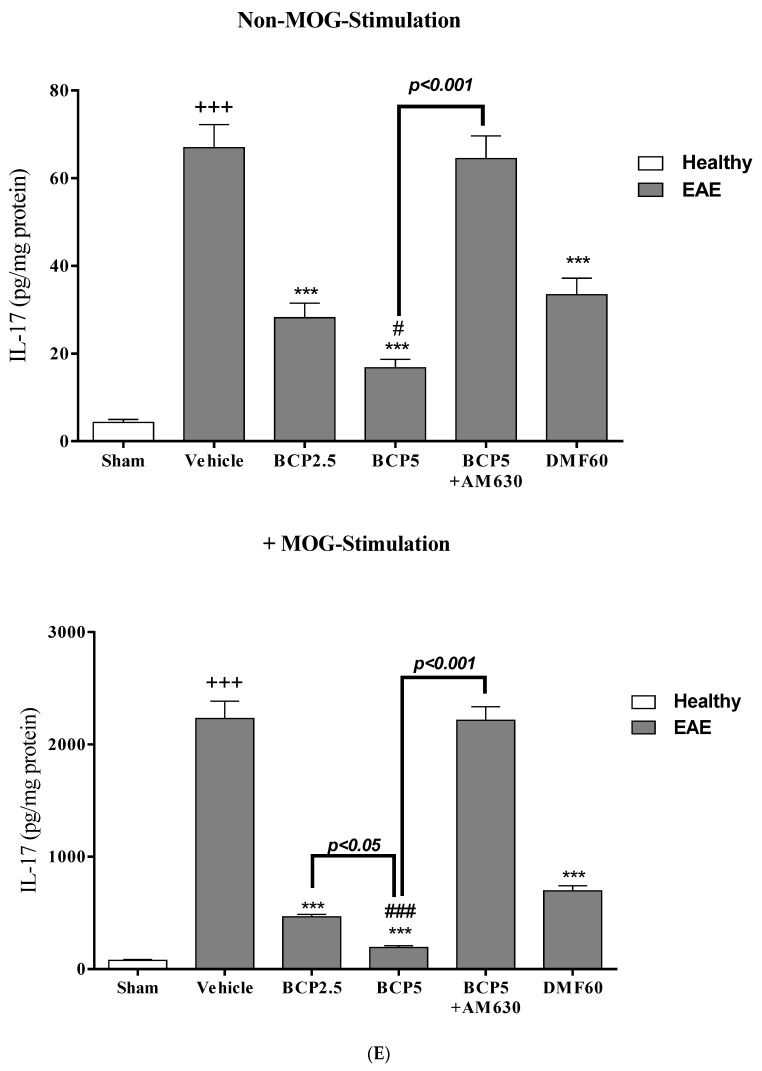

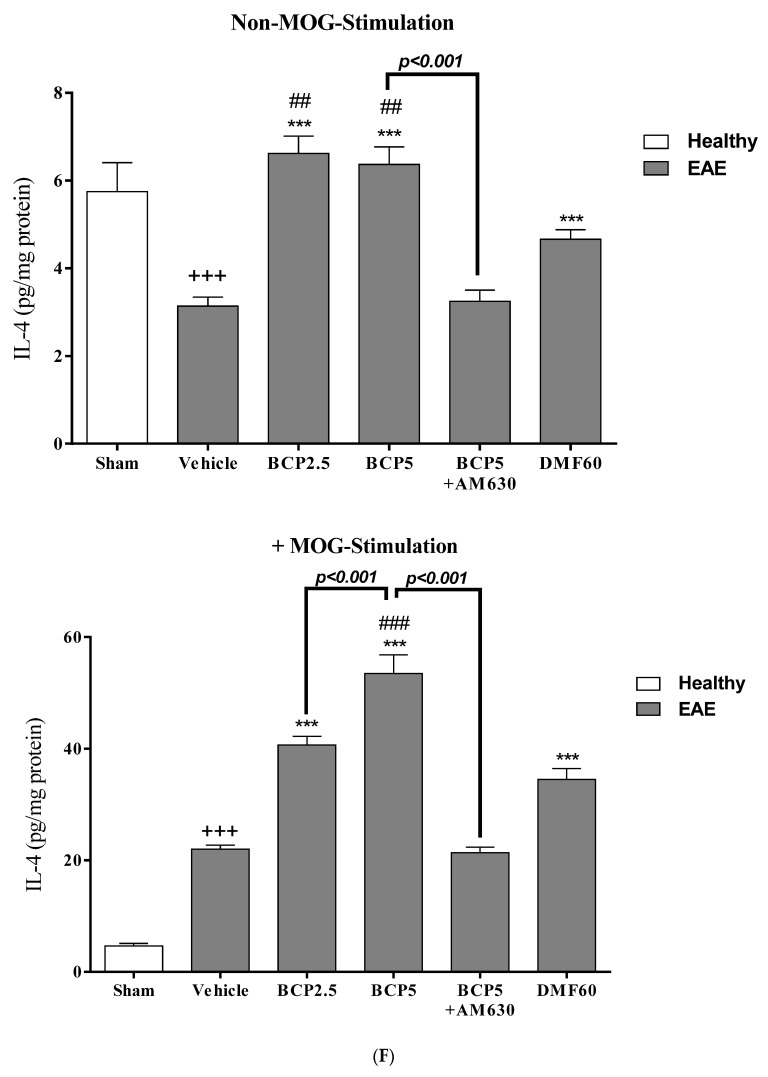

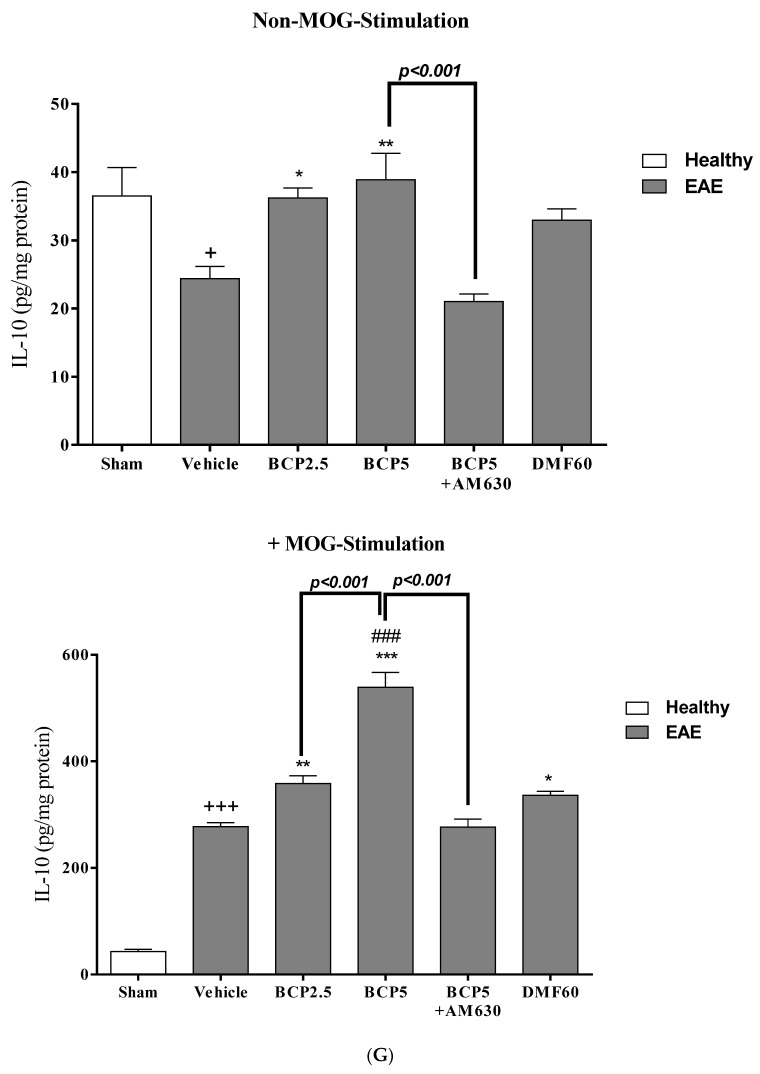

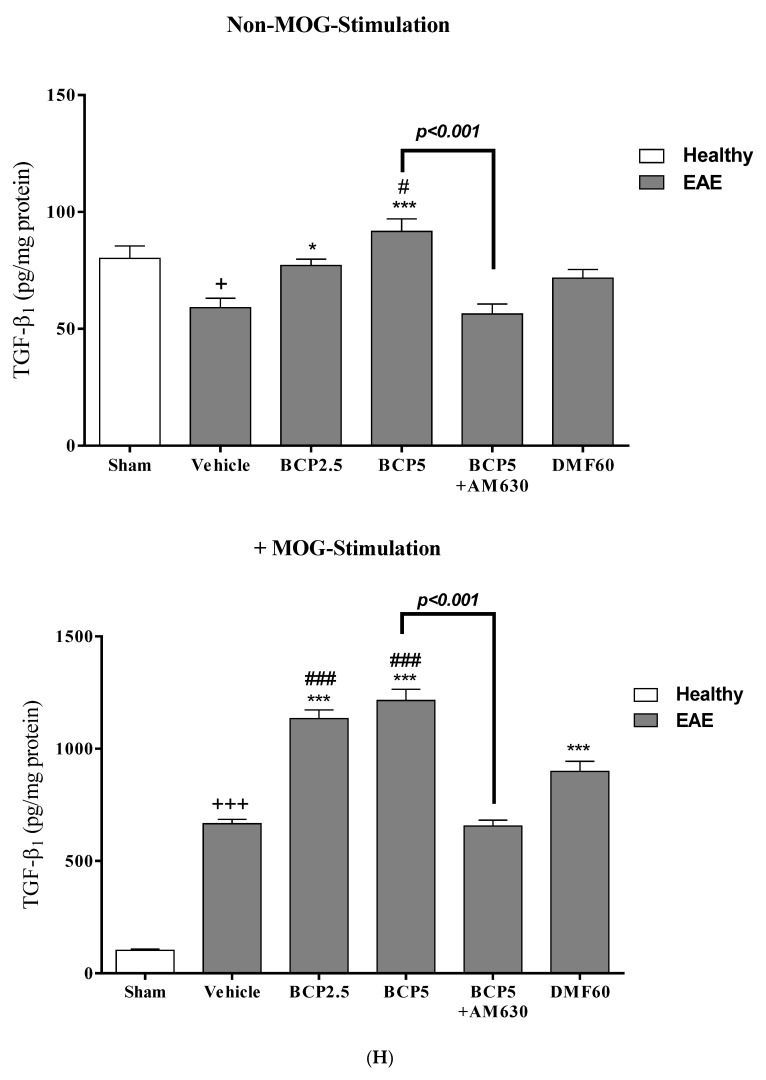

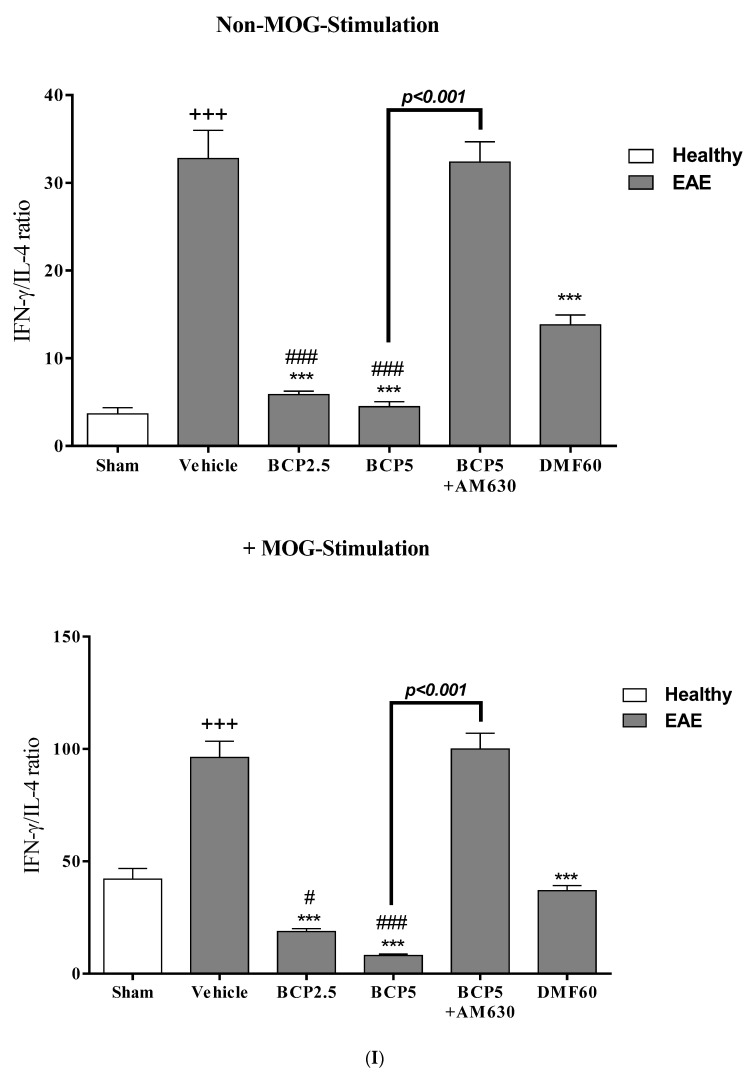

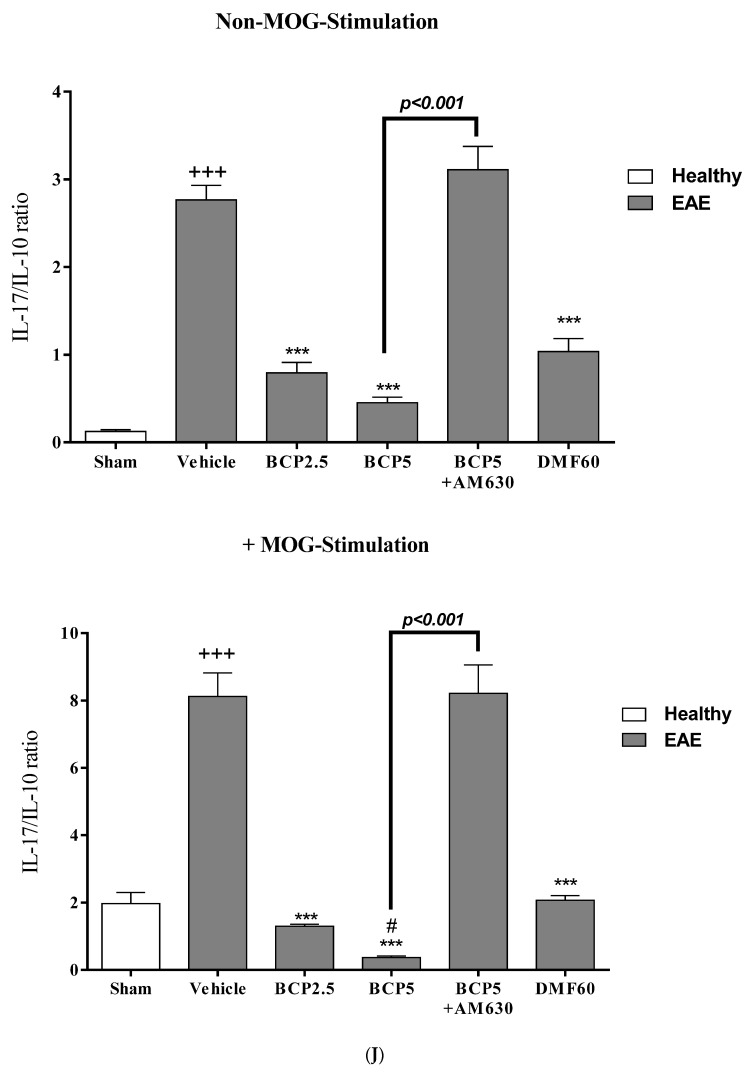

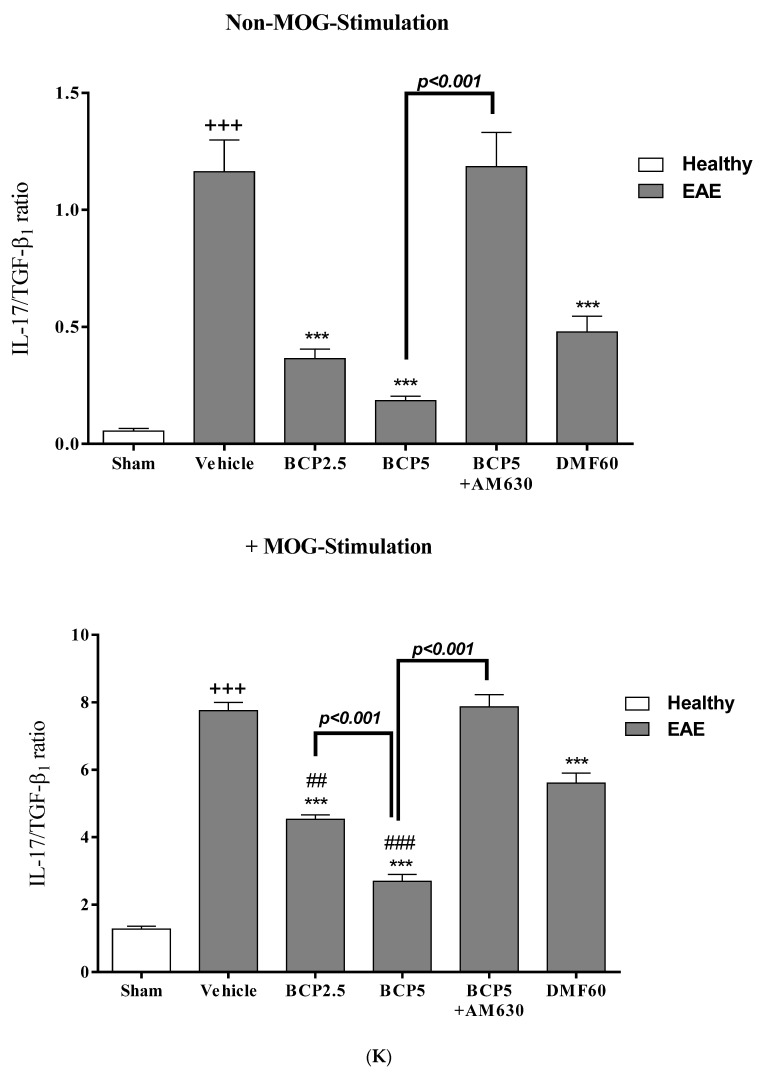
The effects of DMF (60 mg/kg; 30 mg/kg twice a day; p.o.), BCP (2.5, 5 mg/kg/day; p.o.), and the combination of a CB_2_ antagonist AM630 (1 mg/kg; i.p.) with BCP (5 mg/kg/day; p.o.) on cell proliferation, levels of anti- and pro-inflammatory cytokines in spleen lymphocytes of EAE mice; (**A**) Cell proliferation; (**B**) TNF-α; (**C**) IL-6; (**D**) IFN-γ; (**E**) IL-17; (**F**) IL-4; (**G**) IL-10; (**H**) TGF-β_1_; (**I**) ratio of IFN-γ/IL-4; (**J**) IL-ratio of 17/IL-10; and (**K**) ratio of IL-17/ TGF-β_1_. Data were presented as Mean ± SEM, n = six animals per group for each experiment protocol. Two-way ANOVA and Tukey’s multiple comparisons test were performed. (*) indicates the comparison between varying doses of BCP and DMF groups to the vehicle group, *: *p* < 0.05, **: *p* < 0.01, and ***: *p* < 0.001; (^+^) makes the comparison between the vehicle and sham groups, ^+^: *p* < 0.05 and ^+++^: *p* < 0.001; ^#^ in comparison with to DMF group in each graph, ^#^: *p* < 0.05, ^##^: *p* < 0.01, and ^###^: *p* < 0.001.

**Figure 6:**
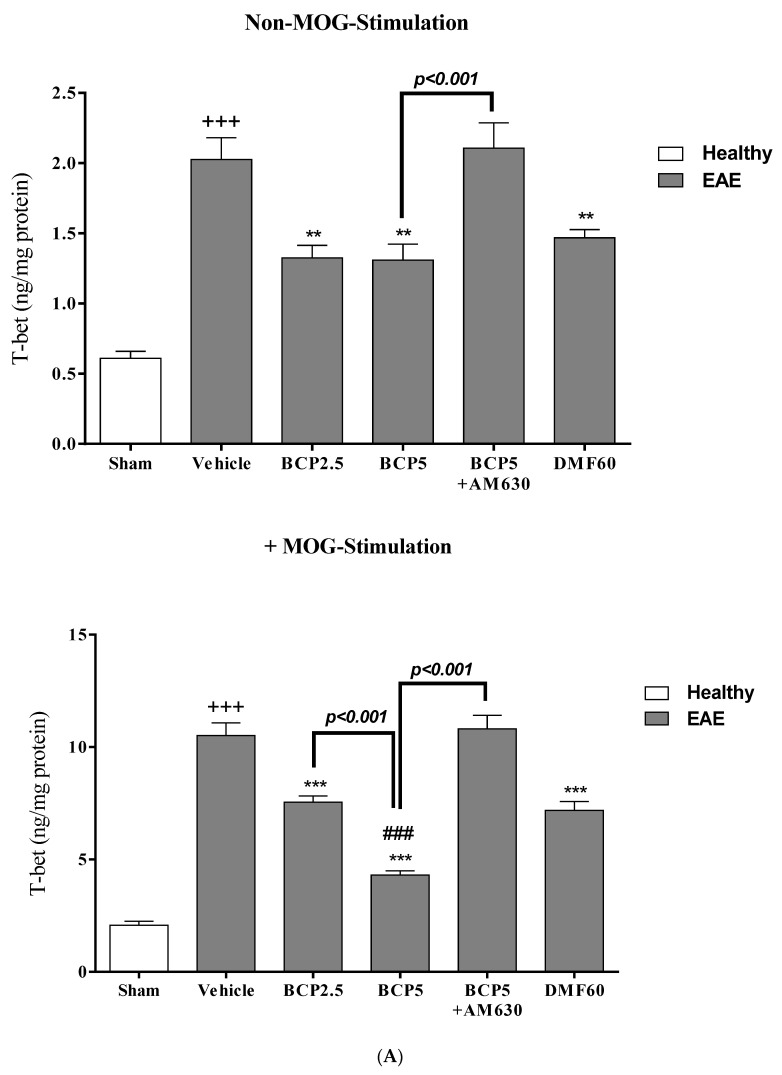

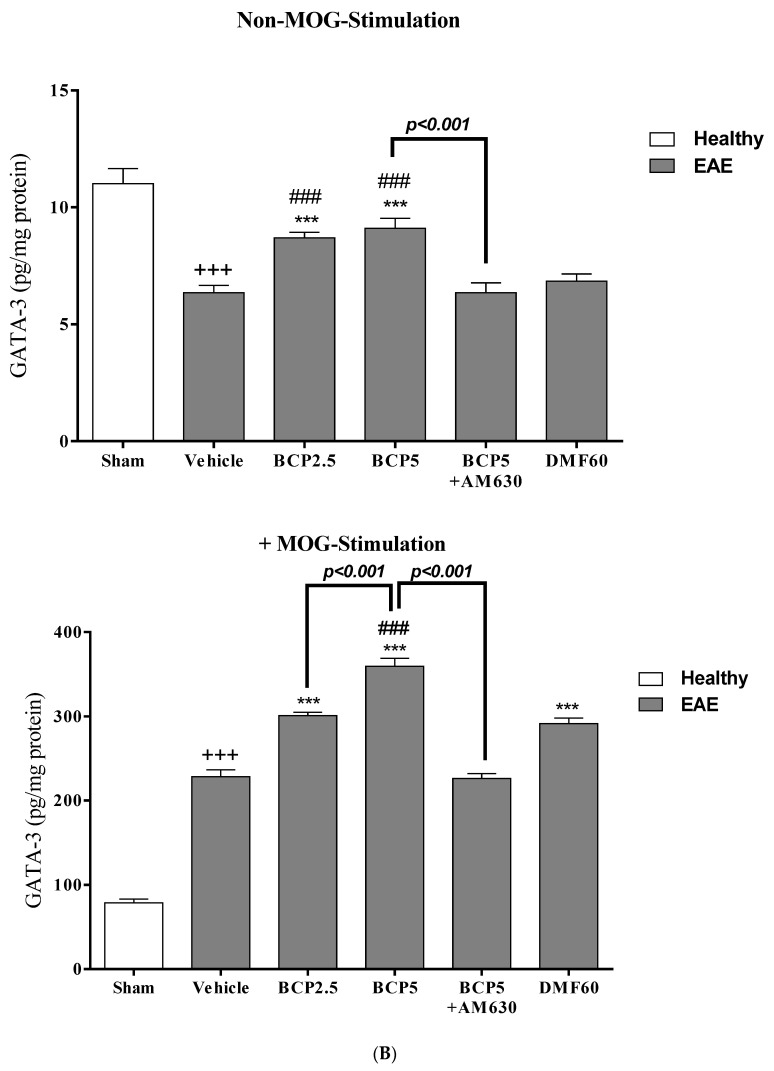

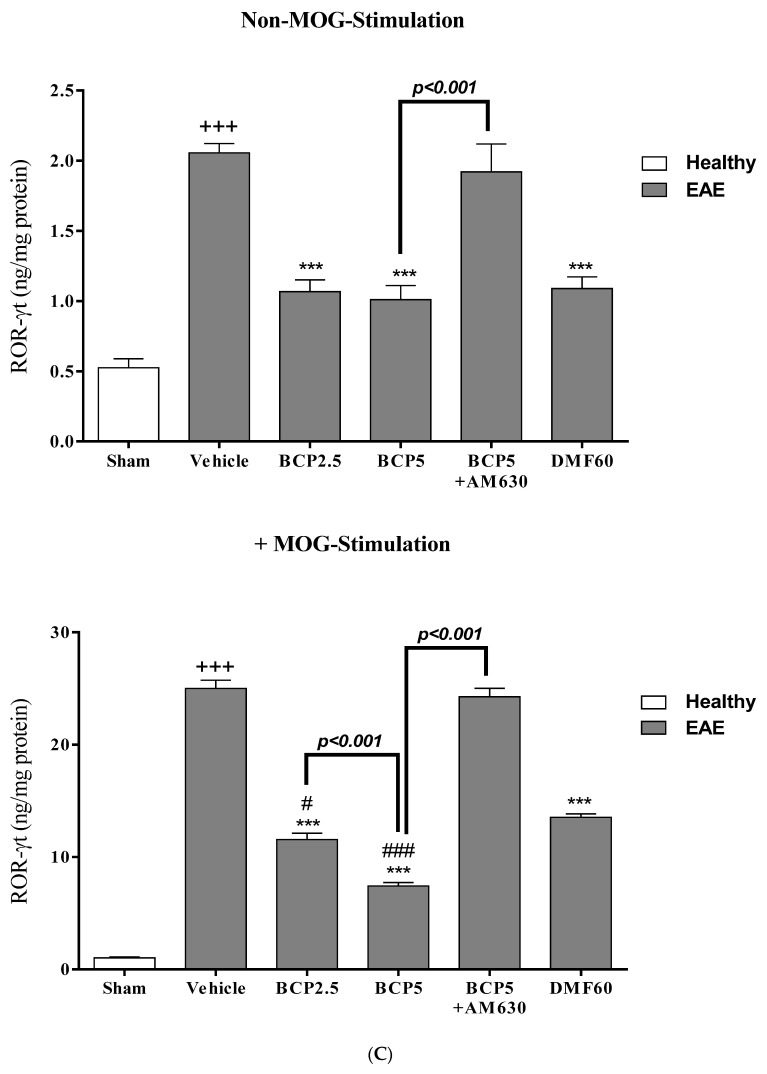

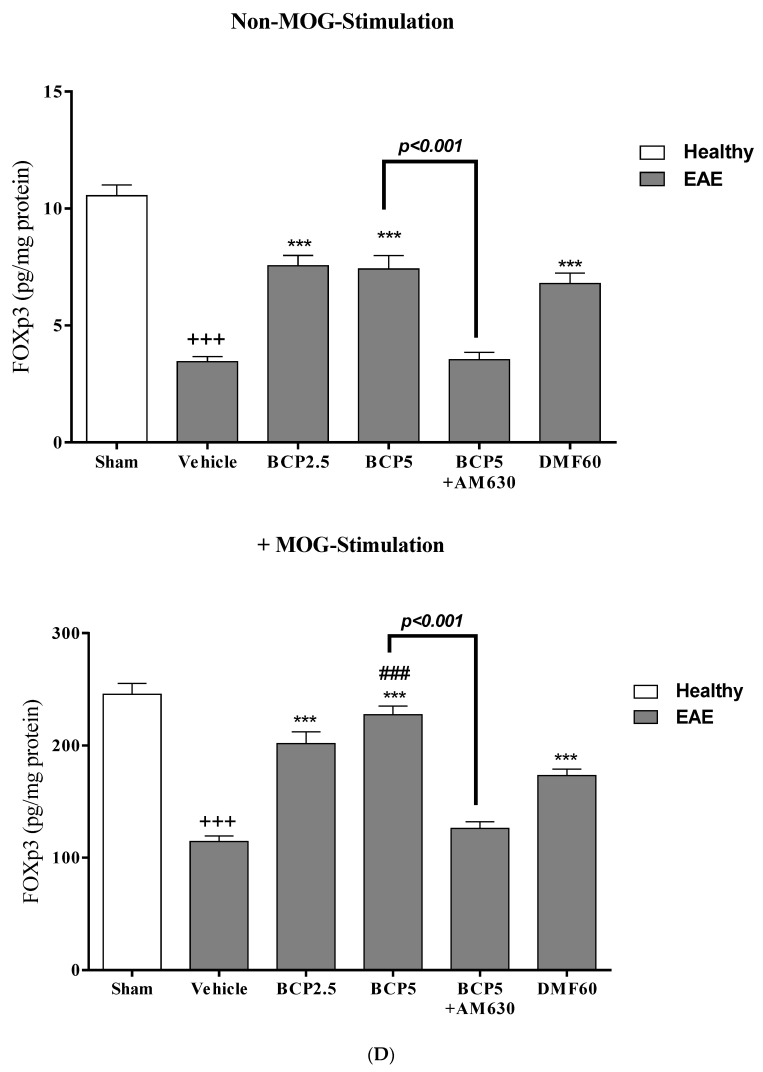

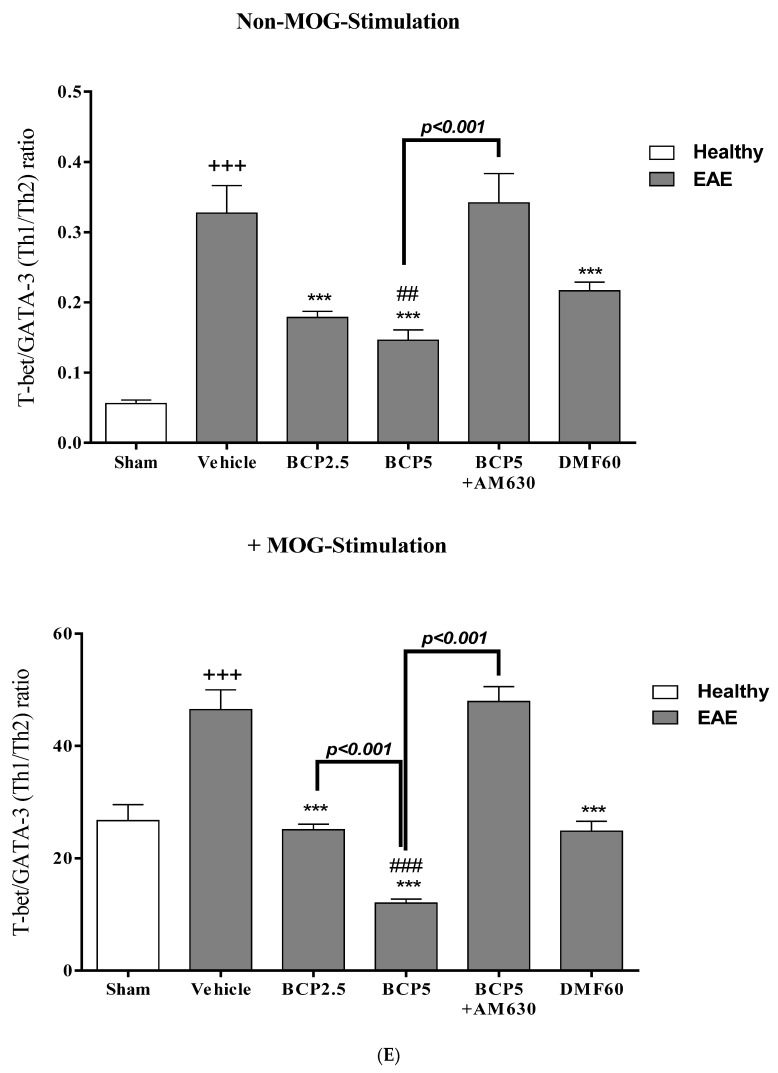

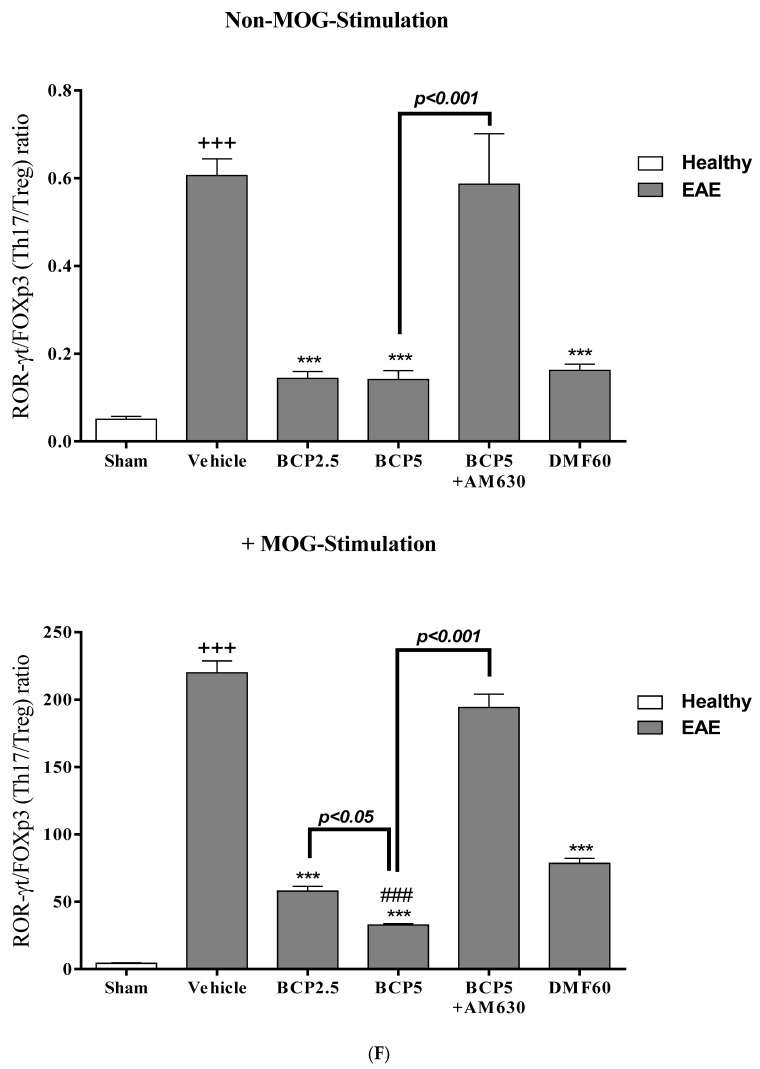
The effects of DMF (60 mg/kg; 30 mg/kg twice a day; p.o.), BCP (2.5, 5 mg/kg/day; p.o.), and the combination of a CB_2_ antagonist AM630 (1 mg/kg; i.p.) with BCP (5 mg/kg/day; p.o.) on the levels of transcription factors, including GATA3 (Th_2_), T-bet (Th_1_), Foxp3 (T_reg_), and ROR-γt (Th_17_) of lymphocytes isolated from EAE mice; (**A**) T-bet; (**B**) GATA3; (**C**) ROR-γt; (**D**) Foxp3; (**E**) ratio of T-bet/GATA3 (Th_1_/Th_2_); and (**F**) ratio of ROR-γt/Foxp3 (Th_17_/T_reg_). Data were presented as Mean ± SEM, n = six animals per group for each experiment protocol. Two-way ANOVA and Tukey’s multiple comparisons test were performed. (*) indicates the comparison between varying doses of BCP or DMF groups to the vehicle group, **: *p* < 0.01 and ***: *p* < 0.001; (^+^) makes a comparison between the vehicle and sham groups, ^+++^: *p* < 0.001; ^#^ compared to DMF groups in each graph, ^#^: *p* < 0.05, ^##^: *p* < 0.01 and ^###^: *p* < 0.001.

When MOG stimulation is absent, the cell proliferation levels (*p* < 0.001, Figure 5A and Figure 6A) and inflammatory cytokines, such as IL-6 (*p* < 0.001, Figure 5C), TNF-α (*p* < 0.001, Figure 5B), IL-17 (*p* < 0.001, Figure 5E), and IFN-γ (*p* < 0.001, Figure 5D) in vehicle EAE group were more significant than the sham group. In contrast, the anti-inflammatory cytokine levels, such as IL-10 (*p* < 0.05, Figure 5G), TGF-β_1_ (*p* < 0.05, Figure 5H), and IL-4 (*p* < 0.001, Figure 5F) in the vehicle EAE group were considerably lower than the sham group. Nevertheless, the vehicle-treated EAE mice group showed lower ratios of the IL-17/IL-10 (*p* < 0.001, Figure 5J), IL-17/TGF-β_1_ (*p* < 0.001, Figure 5K), and IFN-γ/IL-4 (*p* < 0.001, Figure 5I) than the sham group. Additionally, we observed that treatments with BCP (2.5 and 5 mg/kg) and DMF (60 mg/kg) resulted in a reduction in the cell proliferation levels (*p* < 0.001, for all modes, Figure 5A) and levels of inflammatory cytokines, such as IL-6 (*p* < 0.001 for BCP, and *p* < 0.01 for DMF, Figure 5C), TNF-α (*p* < 0.001, for all modes, Figure 5B), IL-17 (*p* < 0.001, for all modes, Figure 5E), and IFN-γ (*p* < 0.001, for all modes, Figure 5D), and the ratios of IL-17/IL-10 (*p* < 0.001, for all modes, Figure 5J), IL-17/TGF-β_1_ (*p* < 0.001, for all modes, Figure 5K), and IFN-γ/IL-4 (*p* < 0.001, for all modes, Figure 5I), compared to vehicle-treated group. Also, BCP treatment (2.5 and 5 mg/kg) caused a significant augmentation in the levels of IL-4 (*p* < 0.001 for both modes, Figure 5F), IL-10 (*p* < 0.05 and 0.01, Figure 5G) and TGF-β_1_ (*p* < 0.05 and 0.001, Figure 5H). When AM630 is present (1 mg/kg), the BCP effects (5 mg/kg) were completely reversed (*p* < 0.001 for all modes, Figure 5A–K).

When the MOG stimulation is present in the vehicle group, we realized that the cell proliferation levels (*p* < 0.001, Figure 5A) and levels of inflammatory cytokines, such as IL-6 (*p* < 0.001, Figure 5C), TNF-α (*p* < 0.001, Figure 5B), IL-17 (*p* < 0.001, Figure 5E), and IFN-γ (*p* < 0.001, Figure 5D), and the ratios of IL-17/IL-10 (*p* < 0.001, Figure 5J), IL-17/TGF-β_1_ (*p* < 0.001, Figure 5K), and IFN-γ/IL-4 (*p* < 0.001, Figure 5I) were markedly elevated compared to the sham group. Conversely, the anti-inflammatory cytokine levels, such as IL-10 (*p* < 0.001, Figure 5G), TGF-β_1_ (*p* < 0.001, Figure 5H), and IL-4 (*p* < 0.001, Figure 5F), in vehicle-treated EAE mice, were lower than the sham group. We additionally found that as a result of treatment with BCP (2.5 and 5 mg/kg) and DMF (60 mg/kg), a significant decline occurred in the cell proliferation levels (*p* < 0.001 for all modes, Figure 5A) and inflammatory cytokine levels, such as IL-6 (*p* < 0.001 for all modes, Figure 5C), TNF-α (*p* < 0.001 for all modes, Figure 5B), IL-17 (*p* < 0.001 for all modes, Figure 5E), and IFN-γ (*p* < 0.001 for all modes, Figure 5D), and the ratios of IL-17/IL-10 (*p* < 0.001 for all modes, Figure 5J), IL-17/TGF-β_1_ (*p* < 0.001 for all modes, Figure 5K), and IFN-γ/IL-4 (*p* < 0.001 for all modes, Figure 5I) were also reduced, while a significant increase was noted in the anti-inflammatory cytokine levels, such as IL-10 (*p* < 0.001 for both doses of BCP, and *p* < 0.05 for DMF, Figure 5G), IL-4 (*p* < 0.001 for all modes, Figure 5F), and TGF-β_1_ (*p* < 0.001 for all modes, Figure 5H), in comparison to vehicle group. Moreover, it was noted in BCP 5 mg/kg group that the cell proliferation levels (*p* < 0.001, Figure 5A) and inflammatory cytokines, such as TNF-α (*p* < 0.01, Figure 5B), IL-6 (*p* < 0.01, Figure 5C), IFN-γ (*p* < 0.001, Figure 5D) and IL-17 (*p* < 0.05, Figure 5E), and the ratio of IL17/TGF-β_1_ (*p* < 0.001, Figure 5K) were considerably lower than BCP 2.5 mg/kg group, while the IL-10 (*p* < 0.001, Figure 5G) and IL4 (*p* < 0.001, Figure 5F) levels were markedly more than BCP 2.5 mg/kg group. The BCP (5 mg/kg) effect on the measured parameters was greater than the DMF group (60 mg/kg) (*p* < 0.001 for all modes except the IL-17/IL-10 ratio with *p* < 0.05, Figure 5A–K). However, 2.5 mg/kg of BCP showed a significant difference in the levels of IL-6 (*p* < 0.05, Figure 5C), TNF-α (*p* < 0.001, Figure 5B), IFN-γ (*p* < 0.001, Figure 5D), TGF-β_1_ (*p* < 0.001, Figure 4H), and the ratios of IL-17/TGF-β_1_ (*p* < 0.01, Figure 5K) and IFN-γ/IL-4 (*p* < 0.05, Figure 5I), in comparison to DMF group (60 mg/kg). When the AM630 (1 mg/kg) was present, the BCP (5 mg/kg) effect on all measured parameters was ultimately declined compared to BCP alone group (*p* < 0.001 for all modes, Figure 5A–K), and had no difference with the vehicle group.

### 3.5. The Effects of BCP on the Levels of Transcription Factors T-Bet (Th_1_), ROR-γt (Th_17_), Foxp3 (T_reg_), and GATA3 (Th_2_) of EAE Mice Lymphocytes with and without MOG Stimulation

Without MOG stimulation, spleen lymphocytes of the vehicle group significantly expressed higher levels of ROR-γt (Th_17_, *p* < 0.001, Figure 6C), T-bet (Th_1_, *p* < 0.001, Figure 6A), and the ratios of ROR-γt/Foxp3 (Th_17_/T_reg_, *p* < 0.001, Figure 6F) and T-bet/GATA3 (Th_1_/Th_2_, *p* < 0.001, Figure 6E) compared to spleen lymphocytes of the sham group. However, the levels of GATA3 (Th_2_, *p* < 0.001, Figure 6B) and Foxp3 (T_reg_, *p* < 0.001, Figure 6D) in the spleen lymphocytes of the vehicle group showed a markedly lower level compared to the sham group. In addition, according to our results, treatment with BCP (2.5 and 5 mg/kg) and DMF (60 mg/kg) showed a reduction in the levels of ROR-γt (*p* < 0.001 for all modes, Figure 6C) and T-bet (*p* < 0.01 for all modes, Figure 6A), and the ratios of ROR-γt/Foxp3 (*p* < 0.001 for all modes, Figure 6F) and T-bet/GATA3 (*p* < 0.001 for all modes, Figure 6E) in spleen lymphocytes compared to vehicle-treated group. Additionally, it was found that levels of Foxp3 (*p* < 0.001 for both cases, Figure 6D) and GATA3 (*p* < 0.001 for both cases, Figure 6B) were markedly increased by BCP (2.5 and 5 mg/kg) in comparison with the vehicle-treated group, while DMF (60 mg/kg) caused a decline in the Foxp3 level (*p* < 0.001, Figure 6D) in comparison to the vehicle group. Our findings also showed that the combination therapy of AM630 (1 mg/kg) with BCP (5 mg/kg) led to a complete blockade of the effects of BCP on transcription factors and their ratios (*p* < 0.001 for all modes, Figure 6A–F).

When the MOG stimulation is present, the levels of GATA3 (Th_2_, *p* < 0.001, Figure 6B), T-bet (Th_1_, *p* < 0.001, Figure 6A), ROR-γt (Th_17_, *p* < 0.001, Figure 6C), and the ratios of ROR-γt/Foxp3 (Th_17_/T_reg_ ratio, *p* < 0.001, Figure 6F) and T-bet/GATA3 (Th_1_/Th_2_ ratio, *p* < 0.001, Figure 6E) were significantly elevated in the vehicle group in comparison with the sham group. On the contrary, the vehicle group showed a significantly lower Foxp3 (T_reg_) level compared to the sham group (*p* < 0.001, Figure 6D). Treatment with BCP (2.5 and 5 mg/kg) and DMF (60 mg/kg) dramatically attenuated the levels of ROR-γt (*p* < 0.001 for all modes, Figure 6C) and T-bet (*p* < 0.001 for all modes, Figure 6A), and the ratios of T-bet/GATA3 (*p* < 0.001 for all modes, Figure 6E) and ROR-γt/Foxp3 (*p* < 0.001 for all modes, Figure 6F) ratios, and significantly increased the levels of Foxp3 (*p* < 0.001 for all modes, Figure 6D) and GATA3 (*p* < 0.001 for all modes, Figure 6B) in comparison with the vehicle group. Also, our findings demonstrated that there was a significant difference in BCP (5 mg/kg)-treated group on the measured parameters in comparison to BCP (2.5 mg/kg)-treated group (*p* < 0.001 for T-bet, GATA3, ROR-γt, and T-bet/GATA3 ratio, *p* < 0.05 for ROR-γt/Foxp3 ratio, Figure 6A–C,E,F). In addition, we realized that the BCP (5 mg/kg) effects on the levels of GATA3 (*p* < 0.001, Figure 6B), T-bet (*p* < 0.001, Figure 6A), Foxp3 (*p* < 0.001, Figure 6D), and ROR-γt (*p* < 0.001, Figure 6C), and the ratios of ROR-γt/Foxp3 (*p* < 0.001, Figure 6F) and T-bet/GATA3 (*p* < 0.001, Figure 6E) were significantly higher than the DMF (60 mg/kg) group. Moreover, we found that the BCP (5 mg/kg) effects were abolished entirely when using AM630 (1 mg/kg) 30 min before the treatment with BCP (*p* < 0.001 for all modes, Figure 6A–F).

### 3.6. The BCP Effects on Cell Proliferation, Cytokines Profile, and Markers of Microglia of EAE Mice with and without MOG Stimulation

**Figure 7:**
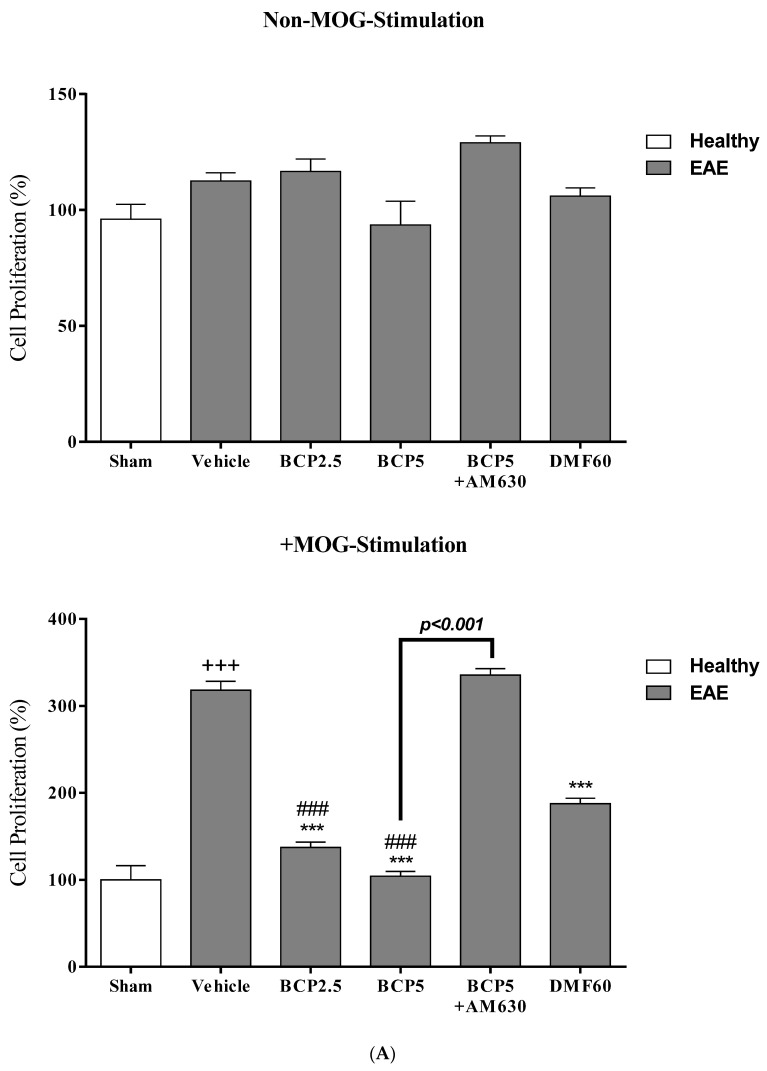

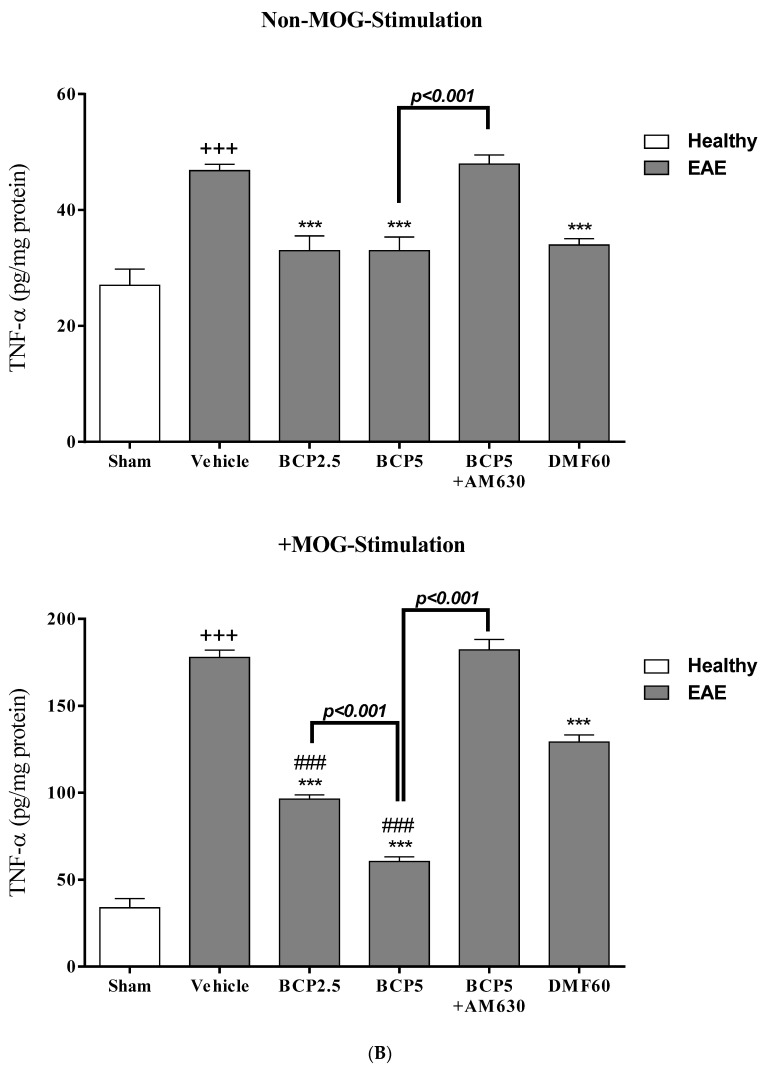

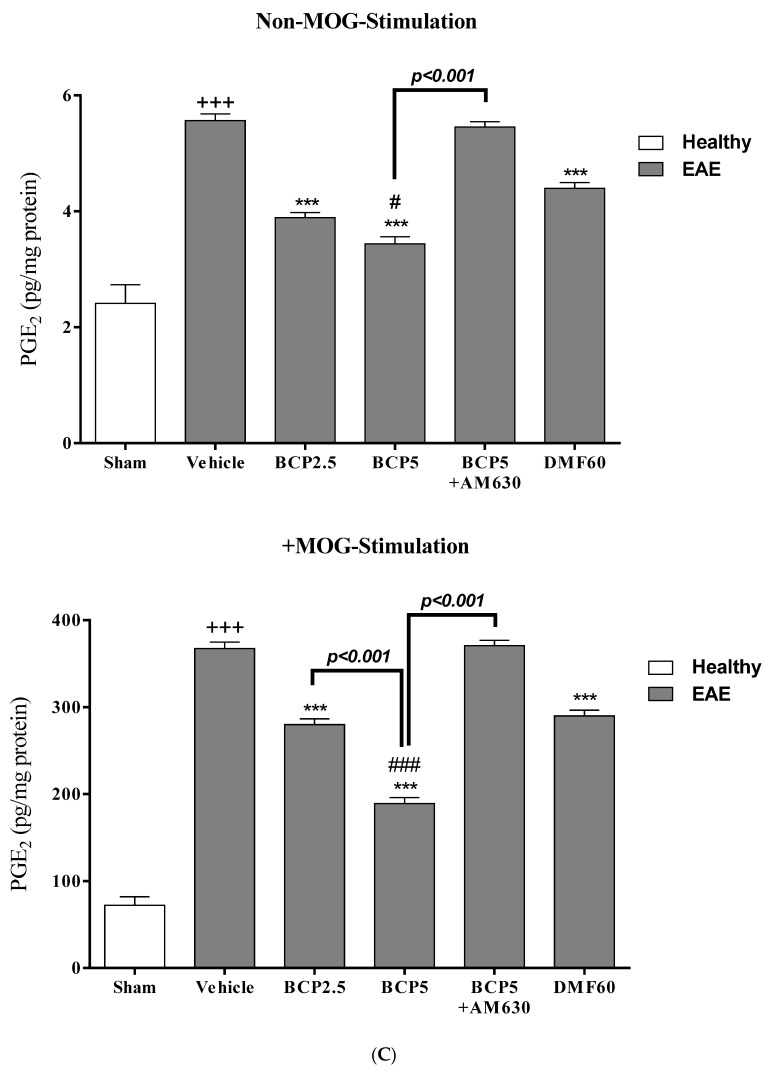

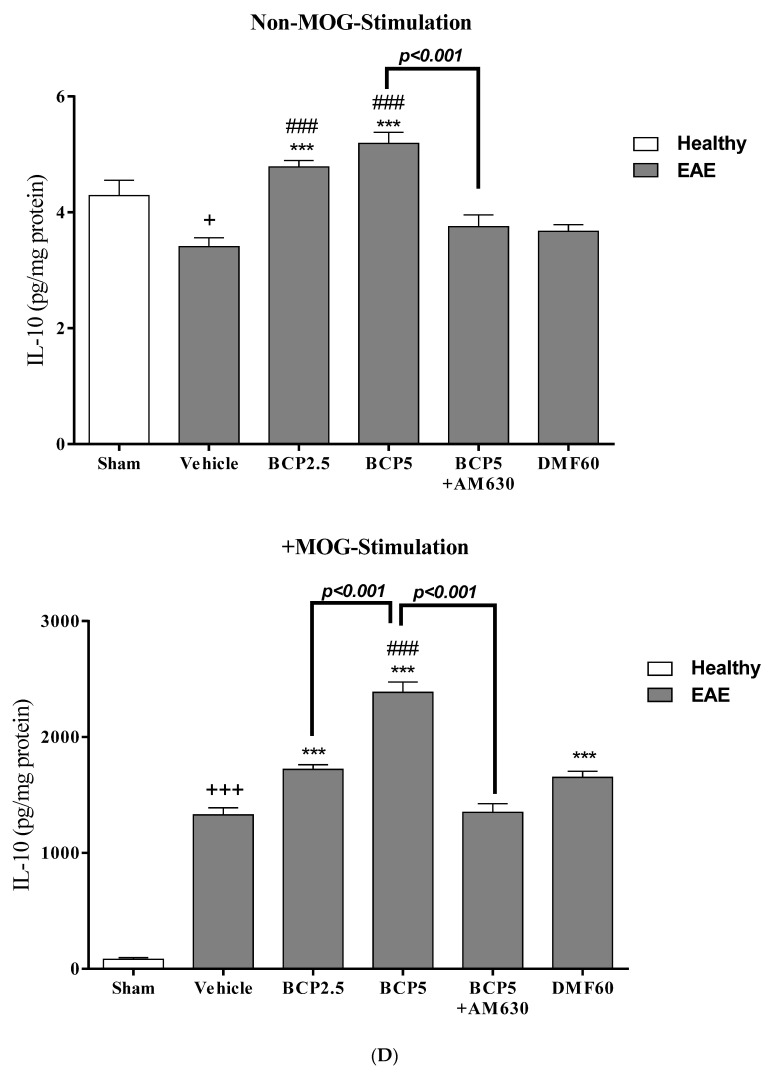
The effects of DMF (60 mg/kg; 30 mg/kg twice a day; p.o.), BCP (2.5, 5 mg/kg/day; p.o.), and the combination of a CB_2_ antagonist AM630 (1 mg/kg; i.p.) with BCP (5 mg/kg/day; p.o.) on the levels of cell proliferation, anti-inflammatory and inflammatory cytokines of microglia separated from EAE mice; (**A**) Cell proliferation; (**B**) TNF-α; (**C**) PGE_2_; and (**D**) IL-10. Data were presented as Mean ± SEM, n = six animals per group for each experiment protocol. Two-way ANOVA and Tukey’s multiple comparisons test were performed. (*) indicates a comparison between varying doses of BCP or DMF groups to the vehicle group, ***: *p* < 0.001; (^+^) makes a comparison between the vehicle and sham groups, ^+^: *p* < 0.05 and ^+++^: *p* < 0.001; ^#^ in comparison to DMF group in each graph, ^#^: *p* < 0.05 and ^###^: *p* < 0.001.

**Figure 8:**
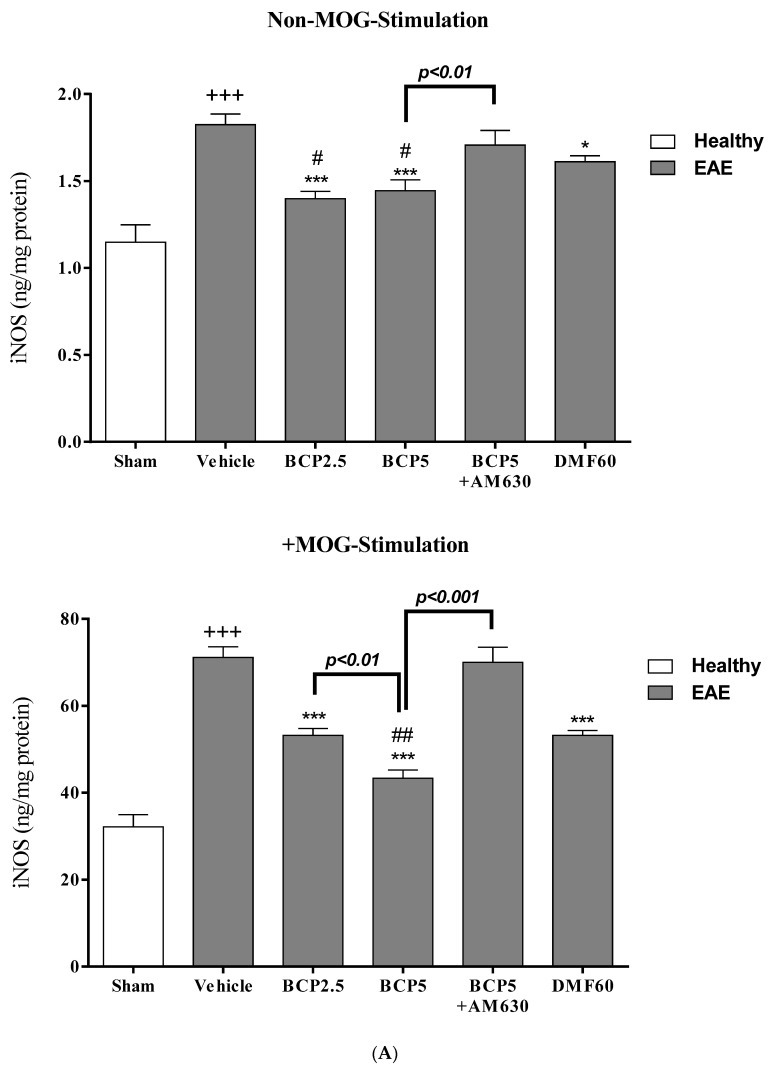

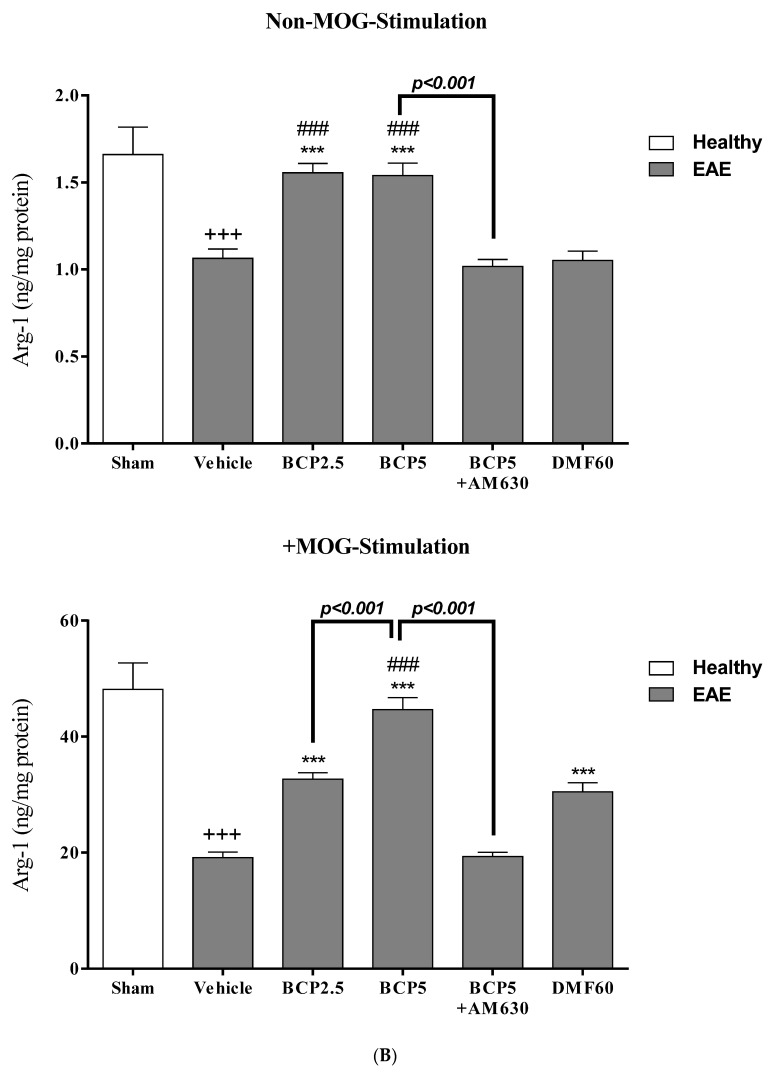

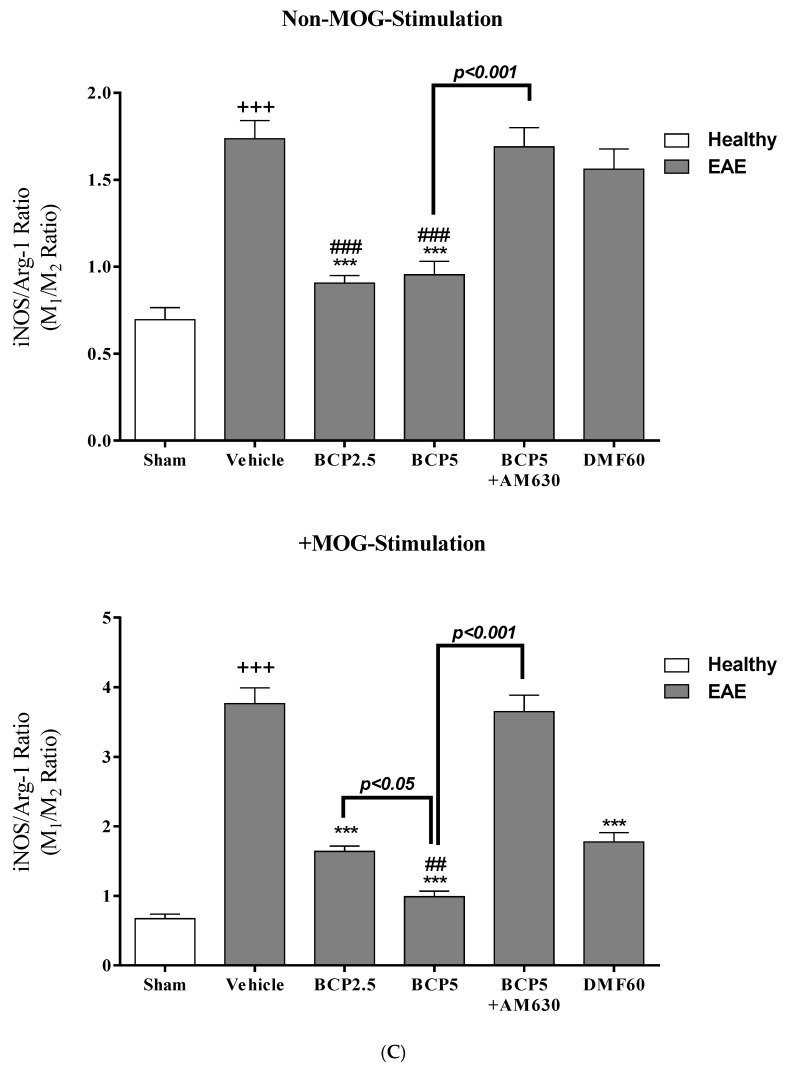

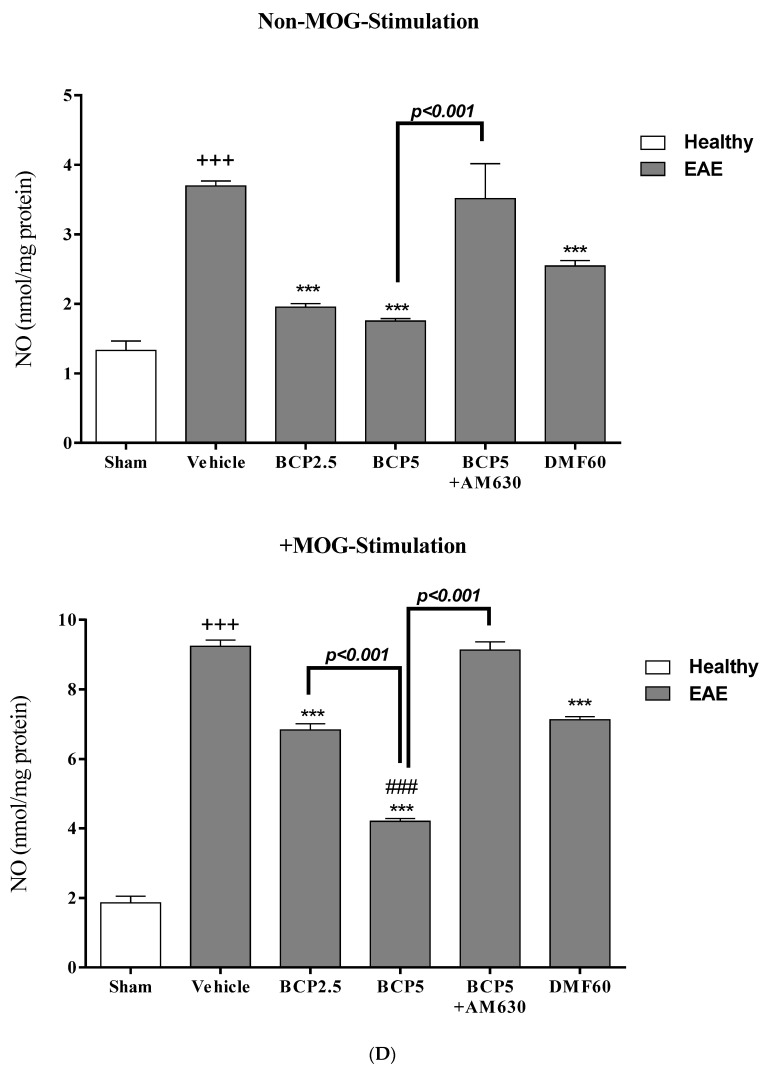

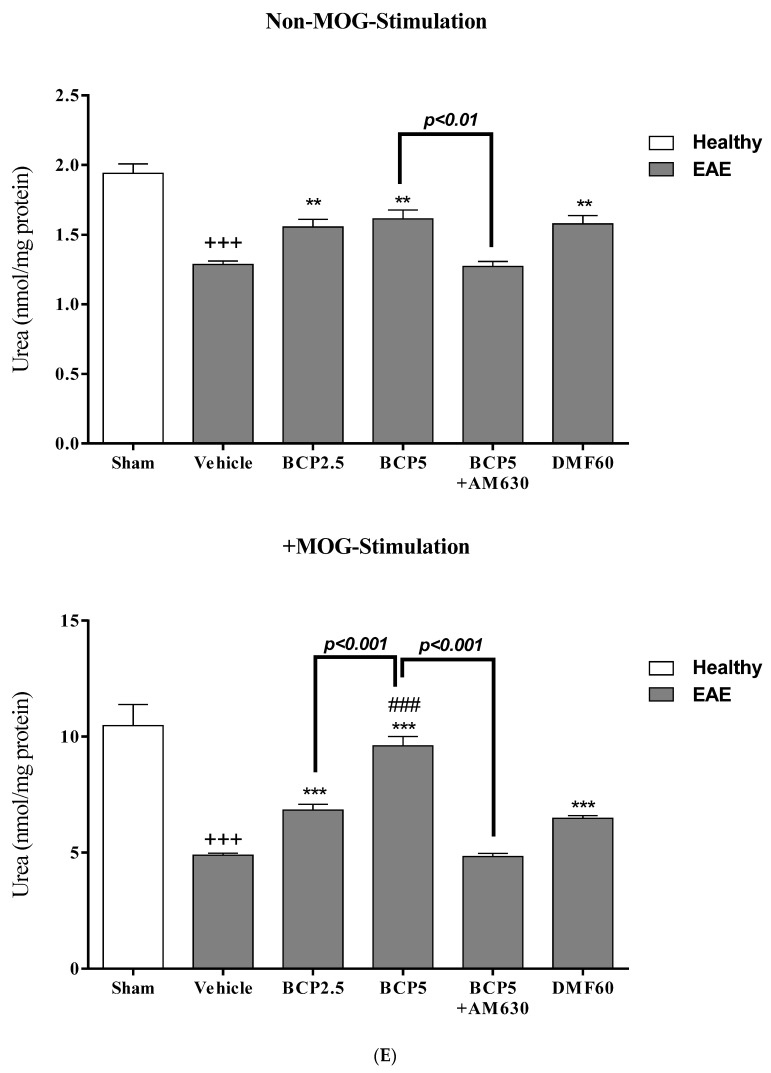

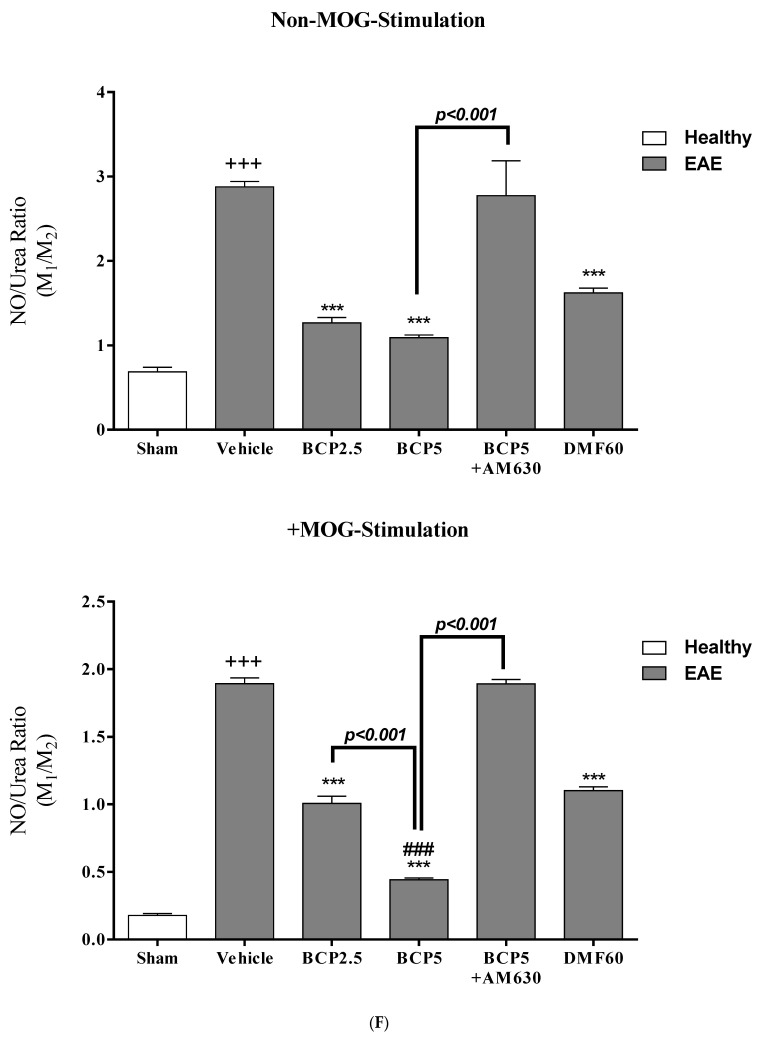
The effects of DMF (60 mg/kg; 30 mg/kg twice a day; p.o.), BCP (2.5, 5 mg/kg/day; p.o.), and the combination of a CB_2_ antagonist AM630 (1 mg/kg; i.p.) with BCP (5 mg/kg/day; p.o.) on intracellular levels of Arg-1 and iNOS and their products, urea, and NO of microglia isolated from EAE mice; (**A**) iNOS; (**B**) Arg-1; (**C**) ratio of iNOS/Arg-1 (M_1_/M_2_); (**D**) NO; (**E**) Urea; (**F**) ratio of NO/Urea (M_1_/M_2_). Data were presented as Mean ± SEM, n = six animals per group for each experiment protocol. Two-way ANOVA and Tukey’s multiple comparisons test were performed. (*) indicates the comparison between varying doses of BCP or DMF groups to the vehicle group, *: *p* < 0.05, **: *p* < 0.01, and ***: *p* < 0.001; (^+^) makes a comparison between the vehicle and sham groups, ^+++^: *p* < 0.001; ^#^ in comparison to DMF group in each graph, ^#^: *p* < 0.05, ^##^: *p* < 0.01, and ^###^: *p* < 0.001.

In non-MOG stimulation, we observed that the levels of PGE_2_ (*p* < 0.001, Figure 7C), TNF-α (*p* < 0.001, Figure 7B), NO (*p* < 0.001, Figure 8D), and iNOS (*p* < 0.001, Figure 8A), and the ratios of NO/urea (*p* < 0.001, Figure 8F) and iNOS/Arg-1 (*p* < 0.001, Figure 8C) in the vehicle-treated group were markedly greater than the sham group. On the contrary, the sham group showed significantly lower levels of Arg-1 (*p* < 0.001, Figure 8B), IL-10 (*p* < 0.05, Figure 7D), and urea (*p* < 0.001, Figure 8E) compared to the vehicle group. Treating EAE animals with DMF (60 mg/kg) significantly reduced the levels of PGE_2_ (*p* < 0.001, Figure 7C), TNF-α (*p* < 0.001, Figure 7B), NO (*p* < 0.001, Figure 8D), iNOS (*p* < 0.05, Figure 8A), and the ratio of NO/urea (*p* < 0.001, Figure 8F), while indicating a significant elevation in the urea level (*p* < 0.01, Figure 8E) in comparison to the vehicle-treated group. Treating EAE animals with BCP (2.5 and 5 mg/kg) dramatically abolished the levels of PGE_2_ (*p* < 0.001 for both modes, Figure 7C), TNF-α (*p* < 0.001 for both modes, Figure 7B), NO (*p* < 0.001 for both modes, Figure 8D), and iNOS (*p* < 0.001 for both modes, Figure 8A), and the ratios of NO/urea (*p* < 0.001 for both modes, Figure 8F) and iNOS/Arg-1 (*p* < 0.001 for both modes, Figure 8C), while causing a significant elevation in the levels of Arg-1 (*p* < 0.001 for both modes, Figure 8B) IL-10 (*p* < 0.001 for both modes, Figure 7D), and urea (*p* < 0.001 for both modes, Figure 8E), compared to the vehicle group. Nevertheless, in the presence of AM630 (1 mg/kg), the BCP (5 mg/kg) effects on all measured parameters were altogether declined (*p* < 0.001 for all modes, Figure 7B–D and Figure 8A–F) compared to the BCP group (5 mg/kg) alone.

In the presence of MOG stimulation, it was demonstrated that the cell proliferation levels (*p* < 0.001, Figure 7A), PGE_2_ (*p* < 0.001, Figure 7C), TNF-α (*p* < 0.001, Figure 7B), IL-10 (*p* < 0.001, Figure 7D), NO (*p* < 0.001, Figure 8D), iNOS (*p* < 0.001, Figure 8A), and the ratios of NO/urea (*p* < 0.001, Figure 8F) and iNOS/Arg-1 (*p* < 0.001, Figure 8C) in microglia of the vehicle group were markedly increased, whereas decreasing the urea (*p* < 0.001, Figure 8E) and Arg-1 (*p* < 0.001, Figure 8B) levels, compared to the sham group. On the other hand, treating with BCP (2.5 and 5 mg/kg) and DMF (60 mg/kg) notably alleviated the cell proliferation levels (*p* < 0.001 for all modes, Figure 7A), PGE_2_ (*p* < 0.001 for all modes, Figure 7C), TNF-α (*p* < 0.001 for all modes, Figure 7B), iNOS (*p* < 0.001 for all modes, Figure 8A), iNOS/Arg-1 ratio (*p* < 0.001 for all modes, Figure 8C), NO/urea ratio (*p* < 0.001 for all modes, Figure 8F), and NO (*p* < 0.001 for all modes, Figure 8D), and a significant elevation was noted in the levels of Arg-1 (*p* < 0.001 for all modes, Figure 8B), IL-10 (*p* < 0.01 for DMF and *p* < 0.001 for BCP, Figure 7D), and urea (*p* < 0.001 for all modes, Figure 8E), compared to the vehicle-treated group. Furthermore, a significant difference was found between the effects of BCP (5 mg/kg) to BCP (*p* < 0.05 for iNOS/Arg-1 ratio, 2.5 mg/kg, *p* < 0.01 for iNOS, and *p* < 0.001 for all measured parameters, Figure 7A–D and Figure 8A–F), and DMF (60 mg/kg, *p* < 0.01 for iNOS, *p* < 0.01 for iNOS/Arg-1 ratio, and *p* < 0.001 for all measured parameters, Figure 7A–D and Figure 8A–F). When the AM630 (1 mg/kg) was present, the BCP (5 mg/kg) effects on the cell proliferation levels, TNF-α, PGE_2_, IL-10, iNOS, Arg-1, iNOS/Arg-1 ratio, urea, NO, and the ratio of NO/urea were declined entirely compared to the group that only received BCP (5 mg/kg) (*p* < 0.001 for all modes, Figure 7A–D and Figure 8A–F).

### 3.7. The Relative Effects of BCP on the CNS Immunity and Systemic Immunity Levels in EAE Mice

**Figure 9:**
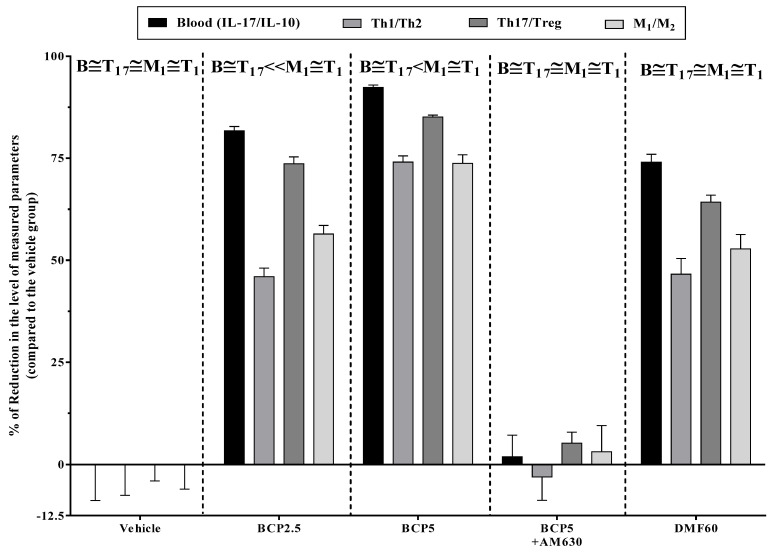
The effects of treatment with BCP on the levels of CNS immunity (microglia) and systemic immunity (blood and spleen lymphocytes) in EAE mice; each column presents the effects of DMF, BCP, and the combination of AM630 and BCP on the decreased levels of anti-inflammatory/ inflammatory ratios in the spleen lymphocytes (Th_17_/T_reg_ and Th_1_/Th_2_), blood (IL-17/IL-10), and microglia (M_1_/M_2_). Data were presented as Mean ± SEM. The B, T_17_, T_1_, and M_1_ Abbreviations represent the ratios of blood IL-17/IL-10, Th_17_/T_reg_, Th_1_/Th_2_ and M_1_/M_2_.

As illustrated in Figure 9, the relative effects of BCP, DMF, and the AM630-BCP combination on the levels of systemic immunity (spleen lymphocytes and blood) and CNS immunity (microglia) were measured as a percentage of reduction of inflammatory/anti-inflammatory ratios in the blood (IL-10/ IL-17) spleen lymphocytes (Th_17_/T_reg_ and Th_1_/Th_2_) and microglia (M_1_/M_2_) compared with the vehicle group in EAE mice. The results showed that BCP (2.5 mg/kg) and DMF (60 mg/kg) declined the Th_17_/T_reg_ levels and blood IL-17/IL-10 ratios more than the Th_1_/Th_2_ levels and M_1_/M_2_ ratios (Figure 9). However, BCP (5 mg/kg) simultaneously targeted both CNS immune system and the systemic system (Figure 9). In contrast, the addition of AM630 before BCP (5 mg/kg) reduced all effects of BCP (5 mg/kg) on the systemic system and CNS immune system (Figure 9).

## 4. Discussion

As far as we know, it is the first work regarding the protective effects of low doses of BCP in treating EAE as a well-established model of chronic MS. In our work, the impacts of low doses of BCP were examined in the presence and absence of a selective pharmacological CB_2_ antagonist (AM630) on the blood cytokine levels, microglia (M_1_/M_2_) polarizations, and lymphocytes (Th_1_/Th_2_ and Th_17_/T_reg_) in EAE mice. As a result, we indicated that low BCP doses significantly decreased clinical and pathological score levels in a dose-dependent fashion, and a selective CB2 antagonist AM630, completely abolished their effects. Indeed, BCP effectively regulated the Th1/Th2 ratios, M1/M2 and Th17/Treg from inflammatory states Th1, Th17, and M1 towards anti-inflammatory conditions Th2, Treg and M2, respectively. Also, according to the results, the BCP effects on the polarizations of lymphocytes and microglia were also strikingly associated with the activation of the CB_2_ receptor since the BCP effects were reversed entirely using CB_2_ antagonist AM630.

Several evidence lines indicate that the EAE model provides inability and inflammation in mice similar to those observed in chronic MS patients. In our study, we demonstrated that EAE mice receiving vehicles represent worsened signatures in the clinical score, body weight, histopathology (H&E staining) and CNS (microglia) and systemic (lymphocytes and blood) inflammations, which are in accordance with the previous studies [48,49,50]. Furthermore, many studies indicated that Th_17_ (expressing ROR-γt and producing IL-17) and Th_1_ (representing T-bet and producing IFN-γ) cells are the primary culprits in the pathogenesis of EAE and MS [5]. In contrast, the anti-inflammatory population of T cells, such as T_reg_ (expressing Foxp3, producing IL-10 and TGF-β) and Th_2_ (expressing GATA3, producing IL-4), results in inflammation resolution [5,51]. In fact, it is worth mentioning that disturbance in ratios of T cells sub-population including Th_17_/T_reg_ and Th_1_/Th_2_ with the propagation of Th_17_ and Th_1_ cells, and their cytokines and transcription factors are recently paid more attention in the pathogenesis of both MS and EAE [51]. Also, infiltration of T cells into the CNS leads to the activation and polarization of microglia toward the inflammatory M_1_ phenotype (producing high levels of inflammatory cytokines and iNOS) [5,52]. Our results also showed that MOG_35–55_ -induced EAE markedly increased the inflammatory cytokine levels in the blood and secreted from spleen lymphocytes and microglial cells. Also, based on the results, the anti-inflammatory cytokine levels secreted from microglia and spleen lymphocytes were notably reduced in this model. Accordingly, the levels of Th_1_/Th_2_, Th_17_/T_reg_, M_1_/M_2_, and blood IL-17/IL-10 ratios shifted towards Th_1_, Th_17_, M_1_ and IL-17 as inflammatory states. In agreement with our findings, several studies also indicated that Th_1_, M_1_, and Th_17_ cells show dominance in the EAE model [53]. In this case, it was demonstrated that MOG_35–55_ -induced EAE provided both systemic and CNS inflammation by elevating the inflammatory cell levels, such as lymphocytes and microglia, respectively [54]. Our results also showed the high sensitivity and specificity of lymphocytes and microglia to MOG_35–55_ peptide stimulation because their proliferation and levels of cytokine were dramatically increased in the MOG stimulation presence.

In the present study, DMF was considered the positive control as an oral medicine approved by FDA for MS treatment. According to findings, DMF (60 mg/kg; p.o.) attenuated the severity of clinical scores and pathological signatures in EAE mice. In addition, the group receiving DMF significantly expressed lower levels of inflammatory cytokines and related transcription factors and higher anti-inflammatory cytokines and related transcription factors. Collectively, DMF caused the shifting of M_1_/M_2,_ Th_1_/Th_2_, and Th_17_/T_reg_ ratios from inflammatory phenotypes toward anti-inflammatory phenotypes T_reg_, M_2_, and Th_2_. Consistent with the present work, various research works described that oral administration of DMF is an effective treatment in different inflammatory and oxidative stress models. In this regard, it has been shown that DMF suppressed Th_1_ and Th_17_ cells differentiation by decreasing the secretion and expression of their pro-inflammatory cytokines, such as TNF-α, IL-17, and IFN-γ [55,56], and expanding the population of the levels of IL-10, IL-5, and IL-4 cytokines and Th_2_ cells [56,57].

Moreover, we demonstrated that DMF treatment (60 mg/kg) mainly targeted blood cytokines and systemic immunity and affected the Th_17_/T_reg_ ratio more than the Th_1_/Th_2_ ratio. Its effects may provide the protective effects of DMF in the EAE model on microglia cells as CNS immunity and systemic immunity. Additionally, we showed that microglia and lymphocytes isolated from EAE mice receiving DMF possessed lower inflammatory cytokine levels and transcription factors in the absence of MOG stimulation than the vehicle group. In agreement with our results, it has been recently reported that patients with MS receiving DMF medication had a persistent change in innate and adaptive immune systems [58]. In this regard, it has been explained that long-term therapy with DMF changes expression patterns of CD4^+^ T cell cytokines by reducing the pro-inflammatory (IFN-γ, GM-CSF, IL-17) and boosting the anti-inflammatory cytokine levels (IL-4, IL-10) [58]. In another study, Parasio and coworkers described the protective effects of DMF on lipopolysaccharide (LPS)-stimulated primary murine microglial cells. Contextually, DMF suppressed the activation of NF-κB and protected neurons against activated microglial cells through both Nrf2-dependent and independent pathways [59]. Peng and colleagues also indicated DMF’s ability to attenuate the levels of inflammatory cytokines, chemokines, and nitric oxide in LPS/IFN-γ-activated microglia and provide healing and anti-inflammatory phenotype M_2_ [60].

Interestingly, we indicated that the low doses of BCP (2.5, 5 mg/kg; p.o.) ameliorated the clinical score, body weight, histopathology (H&E staining), and both CNS (microglia) and systemic (lymphocytes and blood) inflammations, and elevated the levels of anti-inflammatory cytokine IL-10 in EAE mice. Previous works have reported the critical role of microglia and lymphocytes during the neuroinflammatory and neurodegenerative processes in EAE and patients with MS [7,14,15,61], which the regulation of these cells is considered an essential target for the resolution of leukocyte infiltration, inflammation, and disease progression in EAE. We revealed that BCP (2.5, 5 mg/kg) reduced the inflammatory cytokine levels and transcription factors and shifted the imbalanced Th_1_/Th_2_ (IFN-γ/IL-4, T-bet/GATA3), Th_17_/T_reg_ (IL-17/IL-10 and TGF-β_1_, ROR-γt/Foxp3) and M_1_/M_2_ (iNOS/Arg-1, NO/urea) ratios from the inflammatory state to anti-inflammatory Th_2_ (IL-4, GATA3), T_reg_ (IL-10, TGF-β_1_, Foxp3) and M_2_ (Arg-1, urea) state. In fact, BCP exerts its protective effects by reducing the primary culprit cells (Th17 and Th1) involved in the EAE pathogenesis and reactivation of anti-inflammatory cells (M2 and Treg), resulting in the resolution of inflammatory responses. In accordance with our findings, various in vivo and in vitro reported the protective effects of BCP against inflammation.

Additionally, the current work showed that BCP was effective on both CNS immune system (microglia) and systemic system (lymphocytes and blood). However, it seems that BCP simultaneously targets both CNS (microglia) immune systems and systemic (lymphocytes and blood), but we assessed the relative effects of BCP on microglia (M_1_/M_2_) and blood (IL-17/IL-10) lymphocytes (Th_1_/Th_2_ and Th_17_/T_reg_) in the presence of MOG stimulation. Intriguingly, it was illustrated that the effects of BCP at 2.5 mg/kg were different from BCP at 5 mg/kg. Essentially, BCP at 5 mg/kg simultaneously targeted both systemic and CNS immune systems, but BCP at 2.5 mg/kg mainly affected blood cytokines and Th_17_/T_reg_ ratio rather than Th_1_/Th_2_ and M_1_/M_2_ ratios. However, this finding shows that BCP specifically targets Th_17_ and T_reg_ cells, but its effects on CNS immunity cannot be excluded. Recently, the protective effects of BCP on both oligodendrocytes and microglial cells against LPS-induced inflammatory states [14,15] were demonstrated. In this context, we showed that BCP at both high and low concentrations significantly exerted its protective effects via reducing the inflammatory mediator levels (TNF-α, IL-1β, PGE_2_, NO, ROS, iNOS), increasing the anti-inflammatory parameters (Arg-1, IL-10, urea), and polarization of microglial cells to M_2_ anti-inflammatory cells [14], which all these effects were associated in a dose-dependent manner. As determined in our previous works, low BCP concentrations are an efficient therapy against inflammatory and oxidative stress and are also more potent than high concentrations. In agreement with our results, low doses of BCP (1, 5, and 10 mg/kg) impose considerable analgesic effects on pain types in different animal models [62].

Moreover, they showed that BCP (10 mg/kg) attenuates alcoholic steatohepatitis by decreasing inflammation and metabolic dysregulation in mice [63]. These works could support our results about the protective effects of low doses of BCP in EAE as an MS chronic model. Nonetheless, two separate works have reported the efficacy of BCP’s high doses in EAE [25,26]. Contextually, they explained that BCP (25 and 50 mg/kg once a day; p.o. [26], and 25 and 50 mg/kg twice a day; p.o. [25]) had several protective effects on pathological and clinical defects in the EAE model. Additionally, these authors have not tried to show the role of CB_2_ receptors in the protective effects of BCP as a known CB_2_ receptor agonist. As revealed by our previous works, both high and low BCP concentrations exert protective effects via PPAR-γ and Nrf2 receptor signaling pathways depending on the activation of the CB_2_ receptor [14,15]. However, it should take into account that high doses of BCP could provide further nonspecific and adverse effects in the long term of usage, likely through CB_1_ or other involved mechanisms.

Furthermore, another set of experiments was conducted to clarify the role of CB2 receptors in the protective effects of BCP. Interestingly, we demonstrated that BCP results were CB_2_ receptor-mediated since the use of a selective CB_2_ receptor antagonist AM630 entirely disappeared from all protective effects of BCP. Available evidence demonstrated that low concentrations/doses of BCP can exert its protective effects exclusively via the CB_2_ receptor but not CB_1_ [19,62,64,65]. BCP (1, 5, and 10 mg/kg) experimentally provides important relieving effects in animal models of neuropathic and inflammatory pain via CB_2_ receptor due to using a CB2 receptor antagonist or genetical elimination of CB_2_ decreased the protective responses of BCP [62]. Moreover, it was described that using BCP reduces the infiltration and activity of astrocytes and macrophages in the dorsal horn of the spinal cord. Therefore, these effects were all mediated by the CB_2_ receptor [62].

Furthermore, as observed, BCP (10 mg/kg) presents protection for the liver and hinders the over-activation of hepatic macrophage Kupffer cells through their switching toward M_2_ cells. These were the CB_2_ receptor-mediated effects since the CB_2_ receptor ablation entirely inverted the BCP protective effects against binge chronic ethanol-induced inflammation and liver injury [63]. Overall, these research works can approve the efficacy of a low BCP dose in reducing inflammation and demyelination in the spinal cord by hindering the inflammatory cells’ infiltration into the area and polarization of inflammatory macrophages in a CB_2_ receptor-dependent manner.

There are also many studies regarding the protective effects of CB_2_ receptor agonists in preventing and treating EAE. In one study, it has been reported that the CB_2_ receptor activation, but not CB_1_, is mainly required for the therapeutic effect of alpha/beta-hydrolase domain-6 (ABHD6) inhibitor WWL70 in the EAE model because WWL70 did not exert any protective effects in CB_2_ receptor knockout mice or in the presence of a CB_2_ antagonist [66]. Contextually, they found that WWL70 strikingly inhibits the COX-2, iNOS, IL-1β, and TNF-α production and the NF-κB phosphorylation, as well as demyelination and axonal loss. Moreover, amidoalkylindoles derivatives as selective CB_2_ agonists remarkably improve clinical symptoms and protect the CNS against EAE [67]. In another study, a highly selective CB_2_ agonist, Gp1a, ameliorates the clinical scores and reduces the recovery period in EAE, along with the prolonged decrease in demyelination and axonal loss [68]. Our results indicated that Gp1a exerts its protective effects by decreasing pathogenic T cells in the CNS and reducing Th1/Th17 differentiation in peripheral immune organs. On the other hand, CB_2_ receptor agonists provide their protection by decreasing the inflammatory cytokine levels and polarization of M1/Th1/Th17 and increasing the anti-inflammatory cytokine levels and M_2_/Th_2_/T_reg_ polarization [38,68,69]. Also, it has been explained that CB_2_ agonist decreases the level of IL-6 as Th_17_ polarizing cytokine [68,70,71]. Accordingly, we observed that BCP caused a decline in the inflammatory cytokines, such as IL-6, TNF-α, IL-17, IFN-γ and the transcriptional factors of Th_17_ (ROR-γt) and Th_1_ (T-bet), and a significant elevation in the anti-inflammatory cytokines, including TGF-β_1_, IL-10, IL-4, and the transcriptional factors of Th_2_ (GATA3) and T_reg_ (Foxp3), in a dose-dependent fashion. Indeed, the findings exhibited that BCP modulates M_1_/Th_1_/Th_17_ cells towards M_2_/Th_2_/T_reg_, and these effects were tightly associated with activating the CB_2_ receptor since the use of AM630 thoroughly blocked the BCP effects. Moreover, recently, we have shown the functional selectivity of BCP (10^−11^ to 10^−4^ M) by assessing the level of intracellular cAMP in the absence or presence of CB_1_ and CB_2_ receptor antagonists in both oligodendrocytes and microglia models of inflammation [14,15]. Accordingly, BCP dose-dependently reduces the cAMP level and produces a rightward shift by a CB_2_ antagonist. Undoubtedly, it implies that the effects of high and low doses of BCP are both CB_2_ receptor-mediated. The mechanism of protective effects of selective CB_2_ agonists at high doses is different from the signaling mentioned above pathways and depends on the activation of PPAR-γ. Several studies indicated that the ceramide level and PPAR-γ activation signaling pathway are responsible for the effects of higher concentrations/doses of CB_2_ receptor ligands [16,72,73]. Similarly, it has been demonstrated that the protective effects of high concentrations and amounts of BCP (>5 μM and (>25 mg/kg) were mediated via the CB_2_ receptor-PPAR-γ pathway, since the use of PPAR-γ and CB_2_ antagonists totally annihilated the protective effects of BCP [14,15,27,32,74].

In conclusion, according to the present study’s findings, low BCP doses also effectively treated EAE as a chronic MS model. In addition, it was found that the CB2 receptor mediated the protective effects of BCP. Furthermore, our ex vivo and in vivo surveys revealed that BCP decreases the pathological and clinical score in EAE by modulating both adaptive (lymphocytes) and innate (microglia) immune systems from the inflammatory (Th_1_/Th_17_/M_1_) to anti-inflammatory state (Th_2_/T_reg_/M_2_).

## Figures and Tables

**Table 1:** Clinical scoring of EAE mice [28].

Score	Clinical Manifestations (Significant Comment)
**0**	Without clinical symptoms
**1**	Partly limp tail (a **tip of the tail droops, normal gait**)
**2**	Paralyzed tail (**tail droops, normal gait**)
**3**	Uncoordinated movement, hind limb paresis (**uncoordinated gait, hind limbs responding to pinching, tail limps**)
**4**	One paralyzed hind limb (**uncoordinated gait with dragging one hind limb, one hind limb not responding to pinch, tail limps**)
**5**	Two paralyzed hind limbs (**uncoordinated gait with both hind limbs dragging, two hind limbs not responding to pinch, tail limps**)
**6**	Hind limbs paralyzed, weak forelimbs (**uncoordinated gait with forelimbs struggling to pull body, tail limps, forelimbs reflex following pinching**)
**7**	Hind limbs paralyzed, one paralyzed forelimb (no movement of the **mouse, one forelimb responding to a toe pinch, tail limps**)
**8**	Hind limbs paralyzed, both forelimbs paralyzed (**no movement of the mouse, both forelimbs do not respond to a toe pinch, tail limps**)
**9**	Moribund (**worsened breathing, no movement**)
**10**	Death

**Table 2:** The summary of protocols conducted in this research.

Protocol	Groups	EAE	From Day 10 to 37 (Every Day)	
**1** (n = 8/each group)	Sham	-	-	
	Control	✓	Vehicle	
	BCP (2.5 mg/kg)	✓	BCP 2.5 mg/kg/day; p.o.	
	BCP (5 mg/kg)	✓	BCP 5 mg/kg/day; p.o.	
	DMF (60 mg/kg)	✓	DMF 30 mg/kg two times per day; p.o.	
**2** (n = 8/each group)				30 min later
	Sham	-	-	-
	Control	✓	AM630 vehicle; i.p.	+BCP vehicle; p.o.
	BCP (5 mg/kg)	✓	AM630 vehicle; i.p.	+BCP 5 mg/kg/day; p.o.
	AM630 (1 mg/kg) + BCP (5 mg/kg)	✓	AM630 1 mg/kg/day; i.p.	+BCP 5 mg/kg/day; p.o.

**Table 3:** Details for the effects of BCP, DMF, and the combination of BCP+AM630 on the levels of clinical parameters of EAE mice.

Groups	Incidence (Sick/Total)	MOD ^a^	MMCS ^a^	MCS at Peak EAE (Day 17) ^a^	CDI ^a,b^	Day Presents Significant Changes ^c^
Sham	0/16 (0%)	-	0 ± 0	0 ± 0	0	0
Vehicle	16/16 (100%) **^+++^**	6.72 ± 0.42 **^+++^**	7.75 ± 0.47 **^+++^**	7.75 ± 0.47 **^+++^**	29.20 ± 1.45 **^+++^**	9 ^d^
BCP 2.5	7/8 (87.5%)	7.22 ± 0.25	4.00 ± 0.22 ***	3.75 ± 0.18 ***	15.68 ± 1.11 ***	15 ^e^
BCP 5	9/16 (56.25%) ***^, xxx^	6.82 ± 0.52	3.42 ± 0.18 ***^, xxx^	2.75 ± 0.43 ***^, xxx^	10.70 ± 0.83 ***^, xxx^	15 ^e^
BCP 5 + AM630	8/8 (100%) ^∆∆∆^	7.11 ± 0.68	7.55 ± 0.48 ^∆∆∆^	7.35 ± 0.69 ^∆∆∆^	28.38 ± 2.12 ^∆∆∆^	9 ^d^
DMF 60	7/8 (87.5%)	6.52 ± 0.55	4.62 ± 0.21 ***	3.43 ± 0.31 ***	15.81 ± 1.52 ***	15 ^e^

^a^ Data were presented as mean ± SEM. ^b^ Total of clinical scores over the whole period. ^c^ Days after MOG_35–55_ immunization. ^d^ Significant change compared to the sham group. ^e^ Significant change compared to the vehicle group. **^+^** in comparison to the sham group; **^+++^** *p* < 0.001. * in comparison to the vehicle group; *** *p* < 0.001. ^x^ in comparison to DMF group; ^xxx^ *p* < 0.001. ^∆^ comparison of BCP 5 + AM630 group with BCP 5 treated alone group; ^∆∆∆^ *p* < 0.001.

## Data Availability

The data used to support the findings of this study are available on reasonable requests.

## Abbreviations

Analysis of variance (ANOVA), Arginase-1 (Arg-1), Central nervous system (CNS), Cannabinoid receptor (CBR), Cannabinoid receptor type 2 (CB_2_), Complete Freund’s adjuvant (CFA), Cumulative disease index (CDI), Cyclic adenosine monophosphate (cAMP), Dimethyl sulfoxide (DMSO), Dimethyl fumarate (DMF), Dithiothreitol (DTT), Enzyme-linked immunosorbent assay (ELISA), Experimental autoimmune encephalomyelitis (EAE), Fatal bovine serum (FBS), Food and drug administration (FDA), Fluorescein isothiocyanate (FITC), GATA transcription factor 3 (GATA3), Hematoxylin and Eosin (H&E), Hank’s Balanced Salt Solution (HBSS), Inducible nitric oxide synthase (iNOS), Interferon-gamma (IFN-γ), Maximal mean clinical score (MMCS), Interleukin (IL), Mean clinical score (MCS), Mean onset day of disease (MDO), Multiple Sclerosis (MS), MPP^+^ (1-methyl-4-phenylpyridinium), Nuclear factor erythroid 2–related factor 2 (Nrf2), Myelin oligodendrocyte glycoprotein 35–55 (MOG_35–55_), *p* values (*p*), Peroxisome proliferator-activated receptor-gamma (PPAR-γ), Penicillin plus streptomycin (pen/strep), Phenylmethanesulfonyl fluoride (PMSF), Potassium chloride (KCl), Prostaglandin E2 (PGE_2_), Regulatory T cells (T_reg_), Retinoic acid receptor (RAR), Retinoic acid receptor (RAR)-related orphan receptor gamma (ROR-γt), Sphingomyelinase (SMase), T helper (Th), T-box transcription factor TBX21 (T-bet), T helper type 1 (Th_1_), Tumor necrosis factor alpha (TNF-α) Transforming growth factor beta-1 (TGF-β_1_), World Health Organization (WHO), β-Caryophyllene (BCP).

## References

[1] Dobson R., Giovannoni G. (2019). Multiple sclerosis—A review. Eur. J. Neurol..

[2] Goodin D.S., Aminoff M.J., Boller F., Swaab D.F. (2016). Chapter 11—The epidemiology of multiple sclerosis: Insights to a causal cascade. Handbook of Clinical Neurology.

[3] Greenfield A.L., Hauser S.L. (2018). B-cell Therapy for Multiple Sclerosis: Entering an era. Ann. Neurol..

[4] Grace P.M., Loram L.C., Christianson J.P., Strand K.A., Flyer-Adams J.G., Penzkover K.R., Forsayeth J.R., van Dam A.-M., Mahoney M.J., Maier S.F. (2017). Behavioral assessment of neuropathic pain, fatigue, and anxiety in experimental autoimmune encephalomyelitis (EAE) and attenuation by interleukin-10 gene therapy. Brain Behav. Immun..

[5] Constantinescu C.S., Farooqi N., O′Brien K., Gran B. (2011). Experimental autoimmune encephalomyelitis (EAE) as a model for multiple sclerosis (MS). Br. J. Pharmacol..

[6] Álvarez-Sánchez N., Cruz-Chamorro I., López-González A., Utrilla J.C., Fernández-Santos J.M., Martínez-López A., Lardone P.J., Guerrero J.M., Carrillo-Vico A. (2015). Melatonin controls experimental autoimmune encephalomyelitis by altering the T effector/regulatory balance. Brain Behav. Immun..

[7] Askari V.R., Baradaran Rahimi V., Rezaee S.A., Boskabady M.H. (2018). Auraptene regulates Th1/Th2/TReg balances, NF-κB nuclear localization and nitric oxide production in normal and Th2 provoked situations in human isolated lymphocytes. Phytomedicine.

[8] Fernández-Ruiz J., Moreno-Martet M., Rodríguez-Cueto C., Palomo-Garo C., Gómez-Cañas M., Valdeolivas S., Guaza C., Romero J., Guzmán M., Mechoulam R. (2011). Prospects for cannabinoid therapies in basal ganglia disorders. Br. J. Pharmacol..

[9] Lisboa S.F., Gomes F.V., Guimaraes F.S., Campos A.C. (2016). Microglial Cells as a Link between Cannabinoids and the Immune Hypothesis of Psychiatric Disorders. Front. Neurol..

[10] Barrie N., Manolios N. (2017). The endocannabinoid system in pain and inflammation: Its relevance to rheumatic disease. Eur. J. Rheumatol..

[11] Guida F., Luongo L., Boccella S., Giordano M.E., Romano R., Bellini G., Manzo I., Furiano A., Rizzo A., Imperatore R. (2017). Palmitoylethanolamide induces microglia changes associated with increased migration and phagocytic activity: Involvement of the CB2 receptor. Sci. Rep..

[12] Ladak N., Beishon L., Thompson J.P., Lambert D.G. (2011). Cannabinoids and sepsis. Trends Anaesth. Crit. Care.

[13] Dhopeshwarkar A., Mackie K. (2014). CB(2) Cannabinoid Receptors as a Therapeutic Target—What Does the Future Hold?. Mol. Pharmacol..

[14] Askari V.R., Shafiee-Nick R. (2019). The protective effects of β-caryophyllene on LPS-induced primary microglia M1/M2 imbalance: A mechanistic evaluation. Life Sci..

[15] Askari V.R., Shafiee-Nick R. (2019). Promising neuroprotective effects of β-caryophyllene against LPS-induced oligodendrocyte toxicity: A mechanistic study. Biochem. Pharmacol..

[16] Cianchi F., Papucci L., Schiavone N., Lulli M., Magnelli L., Vinci M.C., Messerini L., Manera C., Ronconi E., Romagnani P. (2008). Cannabinoid receptor activation induces apoptosis through tumor necrosis factor alpha-mediated ceramide de novo synthesis in colon cancer cells. Clin. Cancer Res. Off. J. Am. Assoc. Cancer Res..

[17] Rivera I.-G., Ordoñez M., Presa N., Gomez-Larrauri A., Simón J., Trueba M., Gomez-Muñoz A. (2015). Sphingomyelinase D/Ceramide 1-Phosphate in Cell Survival and Inflammation. Toxins.

[18] Mercado N., Kizawa Y., Ueda K., Xiong Y., Kimura G., Moses A., Curtis J.M., Ito K., Barnes P.J. (2014). Activation of transcription factor Nrf2 signalling by the sphingosine kinase inhibitor SKI-II is mediated by the formation of Keap1 dimers. PLoS ONE.

[19] Gertsch J., Leonti M., Raduner S., Racz I., Chen J.Z., Xie X.Q., Altmann K.H., Karsak M., Zimmer A. (2008). Beta-caryophyllene is a dietary cannabinoid. Proc. Natl. Acad. Sci. USA.

[20] Baradaran Rahimi V., Askari V.R. (2022). A mechanistic review on immunomodulatory effects of selective type two cannabinoid receptor beta-caryophyllene. Biofactors.

[21] Wang G., Ma W., Du J. (2018). beta-Caryophyllene (BCP) ameliorates MPP+ induced cytotoxicity. Biomed. Pharmacother..

[22] Hu Y., Zeng Z., Wang B., Guo S. (2017). Trans-caryophyllene inhibits amyloid β (Aβ) oligomer-induced neuroinflammation in BV-2 microglial cells. Int. Immunopharmacol..

[23] Shan J., Chen L., Lu K. (2017). Protective effects of trans-caryophyllene on maintaining osteoblast function. IUBMB Life.

[24] Assis L.C., Straliotto M.R., Engel D., Hort M.A., Dutra R.C., de Bem A.F. (2014). beta-Caryophyllene protects the C6 glioma cells against glutamate-induced excitotoxicity through the Nrf2 pathway. Neuroscience.

[25] Alberti T.B., Barbosa W.L.R., Vieira J.L.F., Raposo N.R.B., Dutra R.C. (2017). (−)-β-Caryophyllene, a CB2 Receptor-Selective Phytocannabinoid, Suppresses Motor Paralysis and Neuroinflammation in a Murine Model of Multiple Sclerosis. Int. J. Mol. Sci..

[26] Fontes L.B.A., Dias D.D.S., Aarestrup B.J.V., Aarestrup F.M., Da Silva Filho A.A., Correa J. (2017). beta-Caryophyllene ameliorates the development of experimental autoimmune encephalomyelitis in C57BL/6 mice. Biomed. Pharmacother..

[27] Bento A.F., Marcon R., Dutra R.C., Claudino R.F., Cola M., Leite D.F., Calixto J.B. (2011). beta-Caryophyllene inhibits dextran sulfate sodium-induced colitis in mice through CB2 receptor activation and PPARgamma pathway. Am. J. Pathol..

[28] Bittner S., Afzali A.M., Wiendl H., Meuth S.G. (2014). Myelin oligodendrocyte glycoprotein (MOG35-55) induced experimental autoimmune encephalomyelitis (EAE) in C57BL/6 mice. J. Vis. Exp. JoVE.

[29] Pitarokoili K., Ambrosius B., Meyer D., Schrewe L., Gold R. (2015). Dimethyl Fumarate Ameliorates Lewis Rat Experimental Autoimmune Neuritis and Mediates Axonal Protection. PLoS ONE.

[30] Kulkarni P., Yellanki S., Medishetti R., Sriram D., Saxena U., Yogeeswari P. (2017). Novel Zebrafish EAE model: A quick in vivo screen for multiple sclerosis. Mult. Scler Relat. Disord..

[31] Javed H., Azimullah S., Haque M.E., Ojha S.K. (2016). Cannabinoid Type 2 (CB2) Receptors Activation Protects against Oxidative Stress and Neuroinflammation Associated Dopaminergic Neurodegeneration in Rotenone Model of Parkinson’s Disease. Front. Neurosci..

[32] Youssef D.A., El-Fayoumi H.M., Mahmoud M.F. (2019). Beta-caryophyllene alleviates diet-induced neurobehavioral changes in rats: The role of CB2 and PPAR-γ receptors. Biomed. Pharmacother..

[33] Al Mansouri S., Ojha S., Al Maamari E., Al Ameri M., Nurulain S.M., Bahi A. (2014). The cannabinoid receptor 2 agonist, beta-caryophyllene, reduced voluntary alcohol intake and attenuated ethanol-induced place preference and sensitivity in mice. Pharmacol. Biochem. Behav..

[34] Askari V.R., Baradaran Rahimi V., Assaran A., Iranshahi M., Boskabady M.H. (2020). Evaluation of the anti-oxidant and anti-inflammatory effects of the methanolic extract of Ferula szowitsiana root on PHA-induced inflammation in human lymphocytes. Drug Chem. Toxicol..

[35] Roohbakhsh Y., Baradaran Rahimi V., Silakhori S., Rajabi H., Rahmanian-Devin P., Samzadeh-Kermani A., Rakhshandeh H., Hasanpour M., Iranshahi M., Mousavi S.H. (2020). Evaluation of the Effects of Peritoneal Lavage with Rosmarinus officinalis Extract against the Prevention of Postsurgical-Induced Peritoneal Adhesion. Planta Med..

[36] Datler H., Vogel A., Kerndl M., Baumgartinger C., Musiejovsky L., Makivic N., Frech S., Niederreiter B., Haider T., Pühringer M. (2019). PI3K activity in dendritic cells exerts paradoxical effects during autoimmune inflammation. Mol. Immunol..

[37] Ernst O., Zor T. (2010). Linearization of the bradford protein assay. J. Vis. Exp. JoVE.

[38] Hegde V.L., Hegde S., Cravatt B.F., Hofseth L.J., Nagarkatti M., Nagarkatti P.S. (2008). Attenuation of experimental autoimmune hepatitis by exogenous and endogenous cannabinoids: Involvement of regulatory T cells. Mol. Pharmacol..

[39] Lee J.K., Tansey M.G. (2013). Microglia isolation from adult mouse brain. Methods Mol. Biol..

[40] Askari V.R., Baradaran Rahimi V., Tabatabaee S.A., Shafiee-Nick R. (2019). Combination of Imipramine, a sphingomyelinase inhibitor, and beta-caryophyllene improve their therapeutic effects on experimental autoimmune encephalomyelitis (EAE). Int. Immunopharmacol..

[41] Shafiee-Nick R., Afshari A.R., Mousavi S.H., Rafighdoust A., Askari V.R., Mollazadeh H., Fanoudi S., Mohtashami E., Rahimi V.B., Mohebbi M. (2017). A comprehensive review on the potential therapeutic benefits of phosphodiesterase inhibitors on cardiovascular diseases. Biomed. Pharmacother..

[42] Ramirez F., Fowell D.J., Puklavec M., Simmonds S., Mason D. (1996). Glucocorticoids promote a TH2 cytokine response by CD4+ T cells in vitro. J. Immunol..

[43] Askari V.R., Rahimi V.B., Zargarani R., Ghodsi R., Boskabady M., Boskabady M.H. (2021). Anti-oxidant and anti-inflammatory effects of auraptene on phytohemagglutinin (PHA)-induced inflammation in human lymphocytes. Pharmacol. Rep..

[44] Baradaran Rahimi V., Mousavi S.H., Haghighi S., Soheili-Far S., Askari V.R. (2019). Cytotoxicity and apoptogenic properties of the standardized extract of Portulaca oleracea on glioblastoma multiforme cancer cell line (U-87): A mechanistic study. EXCLI J..

[45] Curtis M.J., Bond R.A., Spina D., Ahluwalia A., Alexander S.P., Giembycz M.A., Gilchrist A., Hoyer D., Insel P.A., Izzo A.A. (2015). Experimental design and analysis and their reporting: New guidance for publication in BJP. Br. J. Pharmacol..

[46] George C.H., Stanford S.C., Alexander S., Cirino G., Docherty J.R., Giembycz M.A., Hoyer D., Insel P.A., Izzo A.A., Ji Y. (2017). Updating the guidelines for data transparency in the British Journal of Pharmacology—Data sharing and the use of scatter plots instead of bar charts. Br. J. Pharmacol..

[47] Alexander S.P.H., Roberts R.E., Broughton B.R.S., Sobey C.G., George C.H., Stanford S.C., Cirino G., Docherty J.R., Giembycz M.A., Hoyer D. (2018). Goals and practicalities of immunoblotting and immunohistochemistry: A guide for submission to the British Journal of Pharmacology. Br. J. Pharmacol..

[48] Lassmann H., Bradl M. (2017). Multiple sclerosis: Experimental models and reality. Acta Neuropathol..

[49] Van Kaer L., Postoak J.L., Wang C., Yang G., Wu L. (2019). Innate, innate-like and adaptive lymphocytes in the pathogenesis of MS and EAE. Cell. Mol. Immunol..

[50] Lee S.U., Li C.F., Mortales C.L., Pawling J., Dennis J.W., Grigorian A., Demetriou M. (2019). Increasing cell permeability of N-acetylglucosamine via 6-acetylation enhances capacity to suppress T-helper 1 (TH1)/TH17 responses and autoimmunity. PLoS ONE.

[51] Robinson A.P., Harp C.T., Noronha A., Miller S.D. (2014). The experimental autoimmune encephalomyelitis (EAE) model of MS: Utility for understanding disease pathophysiology and treatment. Handb. Clin. Neurol..

[52] Beurel E., Kaidanovich-Beilin O., Yeh W.-I., Song L., Palomo V., Michalek S.M., Woodgett J.R., Harrington L.E., Eldar-Finkelman H., Martinez A. (2013). Regulation of Th1 Cells and Experimental Autoimmune Encephalomyelitis by Glycogen Synthase Kinase-3. J. Immunol..

[53] Wang J., Wang J., Wang J., Yang B., Weng Q., He Q. (2019). Targeting Microglia and Macrophages: A Potential Treatment Strategy for Multiple Sclerosis. Front. Pharmacol..

[54] Liu C., Li Y., Yu J., Feng L., Hou S., Liu Y., Guo M., Xie Y., Meng J., Zhang H. (2013). Targeting the shift from M1 to M2 macrophages in experimental autoimmune encephalomyelitis mice treated with fasudil. PLoS ONE.

[55] Schimrigk S., Brune N., Hellwig K., Lukas C., Bellenberg B., Rieks M., Hoffmann V., Pohlau D., Przuntek H. (2006). Oral fumaric acid esters for the treatment of active multiple sclerosis: An open-label, baseline-controlled pilot study. Eur. J. Neurol..

[56] Wu Q., Wang Q., Mao G. (2017). Dimethyl Fumarate Selectively Reduces Memory T Cells and Shifts the Balance between Th1/Th17 and Th2 in Multiple Sclerosis Patients. J. Immunol..

[57] de Jong R., Bezemer A.C., Zomerdijk T.P., van de Pouw-Kraan T., Ottenhoff T.H., Nibbering P.H. (1996). Selective stimulation of T helper 2 cytokine responses by the anti-psoriasis agent monomethylfumarate. Eur. J. Immunol..

[58] Montes Diaz G., Fraussen J., Van Wijmeersch B., Hupperts R., Somers V. (2018). Dimethyl fumarate induces a persistent change in the composition of the innate and adaptive immune system in multiple sclerosis patients. Sci. Rep..

[59] Paraiso H.C., Kuo P.C., Curfman E.T., Moon H.J., Sweazey R.D., Yen J.H., Chang F.L., Yu I.C. (2018). Dimethyl fumarate attenuates reactive microglia and long-term memory deficits following systemic immune challenge. J. Neuroinflamm..

[60] Peng H., Li H., Sheehy A., Cullen P., Allaire N., Scannevin R.H. (2016). Dimethyl fumarate alters microglia phenotype and protects neurons against pro-inflammatory toxic microenvironments. J. Neuroimmunol..

[61] Lively S., Schlichter L.C. (2018). Microglia Responses to Pro-inflammatory Stimuli (LPS, IFNγ+TNFα) and Reprogramming by Resolving Cytokines (IL-4, IL-10). Front. Cell. Neurosci..

[62] Klauke A.L., Racz I., Pradier B., Markert A., Zimmer A.M., Gertsch J., Zimmer A. (2014). The cannabinoid CB2 receptor-selective phytocannabinoid beta-caryophyllene exerts analgesic effects in mouse models of inflammatory and neuropathic pain. Eur. Neuropsychopharmacol..

[63] Varga Z.V., Matyas C., Erdelyi K., Cinar R., Nieri D., Chicca A., Nemeth B.T., Paloczi J., Lajtos T., Corey L. (2018). beta-Caryophyllene protects against alcoholic steatohepatitis by attenuating inflammation and metabolic dysregulation in mice. Br. J. Pharmacol..

[64] Katsuyama S., Mizoguchi H., Kuwahata H., Komatsu T., Nagaoka K., Nakamura H., Bagetta G., Sakurada T., Sakurada S. (2013). Involvement of peripheral cannabinoid and opioid receptors in beta-caryophyllene-induced antinociception. Eur. J. Pain.

[65] Zhang Z., Yang C., Dai X., Ao Y., Li Y. (2017). Inhibitory effect of trans-caryophyllene (TC) on leukocyte-endothelial attachment. Toxicol. Appl. Pharmacol..

[66] Wen J., Ribeiro R., Tanaka M., Zhang Y. (2015). Activation of CB2 receptor is required for the therapeutic effect of ABHD6 inhibition in experimental autoimmune encephalomyelitis. Neuropharmacology.

[67] Shi Y., Duan Y.H., Ji Y.Y., Wang Z.L., Wu Y.R., Gunosewoyo H., Xie X.Y., Chen J.Z. (2017). Amidoalkylindoles as Potent and Selective Cannabinoid Type 2 Receptor Agonists with in Vivo Efficacy in a Mouse Model of Multiple Sclerosis. J. Med. Chem..

[68] Kong W., Li H., Tuma R.F., Ganea D. (2014). Selective CB2 receptor activation ameliorates EAE by reducing Th17 differentiation and immune cell accumulation in the CNS. Cell. Immunol..

[69] Zhang M., Martin B.R., Adler M.W., Razdan R.J., Kong W., Ganea D., Tuma R.F. (2009). Modulation of cannabinoid receptor activation as a neuroprotective strategy for EAE and stroke. J. Neuroimmune Pharmacol. Off. J. Soc. NeuroImmune Pharmacol..

[70] Zarruk J.G., Fernandez-Lopez D., Garcia-Yebenes I., Garcia-Gutierrez M.S., Vivancos J., Nombela F., Torres M., Burguete M.C., Manzanares J., Lizasoain I. (2012). Cannabinoid type 2 receptor activation downregulates stroke-induced classic and alternative brain macrophage/microglial activation concomitant to neuroprotection. Stroke.

[71] Wang Z.Y., Wang P., Bjorling D.E. (2013). Activation of cannabinoid receptor 2 inhibits experimental cystitis. Am. J. Physiol. Regul. Integr. Comp. Physiol..

[72] Herrera B., Carracedo A., Diez-Zaera M., Gomez del Pulgar T., Guzman M., Velasco G. (2006). The CB2 cannabinoid receptor signals apoptosis via ceramide-dependent activation of the mitochondrial intrinsic pathway. Exp. Cell Res..

[73] Olea-Herrero N., Vara D., Malagarie-Cazenave S., Diaz-Laviada I. (2009). Inhibition of human tumour prostate PC-3 cell growth by cannabinoids R(+)-Methanandamide and JWH-015: Involvement of CB2. Br. J. Cancer.

[74] Cheng Y., Dong Z., Liu S. (2014). beta-Caryophyllene ameliorates the Alzheimer-like phenotype in APP/PS1 Mice through CB2 receptor activation and the PPARgamma pathway. Pharmacology.

